# Circulating exhausted CD8+ effector memory cells differentiate immune checkpoint inhibitor-induced liver injury from other acute immune-mediated liver injuries

**DOI:** 10.1136/jitc-2025-014178

**Published:** 2026-03-27

**Authors:** Stuart Astbury, Edmond Atallah, Jane I Grove, Amber G Bozward, Scott P Davies, Mark J Sheehan, Steven W Kumpf, Jessie Qian, Natalia M Krajewska, Grace E Wootton, Melanie R Lingaya, Davor Kresnik, Flavia Radulescu, Ankit Rao, Hester Franks, Lourdes Ruiz-Ortega, Mar Riveiro-Barciela, Shashi K Ramaiah, Thomas A Lanz, Changhua Ji, Poulam M Patel, Ye H Oo, Guruprasad P Aithal

**Affiliations:** 1 Nottingham Digestive Diseases Centre, Translational Medical Sciences, School of Medicine, University of Nottingham, Nottingham, UK; 2 NIHR Nottingham Biomedical Research Centre, Nottingham University Hospitals NHS Trust and the University of Nottingham, Nottingham, UK; 3 Centre for Liver and Gastrointestinal Research, Institute of Biomedical Research, Institute of Immunology and Immunotherapy, University of Birmingham, Birmingham, UK; 4 NIHR Birmingham Biomedical Research Centre, University Hospitals Birmingham NHS Foundation Trust and the University of Birmingham, Birmingham, UK; 5 Birmingham Advanced Cellular Therapy Facility, University of Birmingham, Birmingham, UK; 6 Drug Safety Research and Development, Pfizer Inc, Groton, Connecticut, USA; 7 Department of Oncology, Nottingham University Hospitals NHS Trust, Nottingham, UK; 8 Centre for Cancer Sciences & Biodiscovery Institute, University of Nottingham, Nottingham, UK; 9 Liver Unit, Department of Internal Medicine, Hospital Universitari Vall d'Hebron, Barcelona, Catalonia, Spain; 10 Department of Medicine, Universitat Autònoma de Barcelona, Barcelona, Catalonia, Spain; 11 Drug Safety Research and Development, Pfizer Inc, Cambridge, Massachusetts, USA; 12 Drug Safety Research and Development, Pfizer Inc, La Jolla, California, USA

**Keywords:** Immune Checkpoint Inhibitor, Autoimmune, T-Lymphocytes, Hepatoxicity

## Abstract

**Background:**

Checkpoint inhibitor-induced liver injury (ChILI) is an immune-related adverse reaction, occurring in patients with cancer receiving immune checkpoint inhibitors (CPI). ChILI is currently managed with high doses of corticosteroids which carry their own risks and potential side effects, and the lack of available biomarkers makes monitoring patients at risk of developing ChILI a challenge. There is no specific test that distinguishes ChILI from other competing diagnoses such as acute autoimmune hepatitis (AIH) and idiosyncratic drug-induced liver injury (DILI) due to other medications.

**Methods:**

Patients with cancer taking immunotherapy who did and did not develop ChILI were recruited. Patients gave samples during the acute phase of liver injury, before CPI treatment and at 12 weeks following the start of CPI therapy if no toxicity developed. Healthy controls were recruited as well as patients with DILI and AIH. Whole blood was taken for broad immune phenotyping using mass cytometry, peripheral blood mononuclear cells were isolated for validatory flow cytometry and single-cell RNA sequencing (RNA-seq), and plasma was used for cytokine profiling. Samples from a second ChILI cohort were used for validation. Snap-frozen liver biopsies were used for bulk RNA-seq to compare the immune response in ChILI to DILI and AIH and correlate with peripheral immune signals. Formalin-fixed paraffin-embedded liver biopsies were used to visualize liver CD8^+^ T-cell infiltration using confocal microscopy.

**Results:**

We have identified a circulating CD8^+^ effector memory T-cell subset, expressing high levels of CD38, HLA-DR and CXCR3 and significantly correlated with alanine transaminase. Flow cytometry and single-cell RNA sequencing revealed an increase in granzyme expression, the liver residency marker CD69 and exhaustion markers CTLA-4, PDCD1 and HAVCR2 relative to other CD8^+^ effector subsets. Liver tissue bulk RNA-seq and immune cell deconvolution showed a significant increase in resident CD8^+^ T cells in ChILI compared with DILI and AIH, and a significant upregulation of genes related to CXCR chemokine receptor binding. Plasma cytokine profiling highlighted soluble CD27 and PD-1 as significantly elevated in ChILI relative to controls on CPI.

**Conclusions:**

We have shown that circulating CD8^+^ T cells provide a potential biomarker to distinguish ChILI from DILI and AIH, and highlight different mechanistic pathways between ChILI and other immune-mediated liver injuries.

**WHAT IS ALREADY KNOWN ON THIS TOPIC**

- With a widening range of cancers suitable for treatment with immune checkpoint inhibitors, the incidence of immune-related adverse events including immune checkpoint inhibitor-induced liver injury (ChILI) is rising. ChILI is treated with high doses of immunosuppressive corticosteroids, which can negatively affect cancer outcomes, reduce quality of life for patients and increase risk of infection. There is no specific non-invasive biomarker to confirm the diagnosis or stratify patients with ChILI, or those at risk of developing it. Activation of CD8^+^ T cells is known to occur in ChILI and other immunotherapy toxicities, but this has not been compared with other immune-mediated liver diseases with very similar clinical presentation.

**WHAT THIS STUDY ADDS**

- Using mass and flow cytometry and single-cell RNA sequencing, we have characterized a population of CD8^+^ T cells unique to ChILI. Using RNA sequencing of liver tissue, we have identified key pathways involved in the immune response during ChILI that are distinct from other immune-mediated liver injuries.

**HOW THIS STUDY MIGHT AFFECT RESEARCH, PRACTICE OR POLICY**

- We highlight previously uncharacterized differences between ChILI and liver injury due to other drugs (idiosyncratic drug-induced liver injury) and autoimmune hepatitis, and propose a number of markers to aid in the early detection of ChILI. The unique cell type we have characterized has the potential to identify patients with ChILI who may require treatment with corticosteroids.

## Introduction

 The use of checkpoint inhibitors (CPI) has exponentially increased following their remarkable efficacy in improving survival in many advanced malignancies. In patients with advanced melanoma or renal cell carcinoma, treatment with CPI targeting programmed cell death protein 1 (PD-1) (eg, nivolumab, pembrolizumab) as monotherapy or in combination with anti-cytotoxic T-lymphocyte-associated protein 4 (CTLA-4) (ipilimumab with nivolumab) is now established regimes as standard of care.^1 2^ CPI work by blocking immune suppressive ligand-receptor interactions, enhancing the anticancer cytotoxic effects of tumor-specific T lymphocytes. However, because checkpoint molecules are also involved in immune tolerance their non-specific inhibition triggers activation of autoreactive T cells and the potential targeting of self-antigens,^3^ resulting in a range of immune-related toxicities affecting many organs including the liver. Checkpoint inhibitor-induced liver injury (ChILI) accounts for an increasing proportion of recent drug-induced liver injury (DILI) cohort studies.^4 5^ ChILI usually occurs after 6–14 weeks of CPI and is among the leading causes of immune-related adverse events (irAE) following checkpoint blockade.^6^ A 10-year retrospective study estimated ChILI occurs in 8.8% of all those treated with CPI, rising to 32% of patients treated with combination therapy.^7^

ChILI presents with features of acute liver injury and therefore shares clinical characteristics with idiosyncratic DILI and acute presentation of autoimmune hepatitis (AIH). Although ChILI presents with a hepatocellular pattern of injury in 54% of cases, this can vary based on the CPI regimen used.^7^ Studies have reported distinctive histological features that may differentiate ChILI from DILI and AIH;^8^ however, only a small fraction of patients with ChILI undergo a liver biopsy,^7^ and there is a lack of peripheral biomarkers.^9^ Peripheral monocyte and CD8^+^ T-cell activation have been demonstrated in ChILI^10^ but have not been profiled in detail or compared with other similarly presenting immune-mediated liver diseases. As patients with cancer are occasionally on multiple medications to treat their comorbidities, including anti-infective agents,^11^ attributing liver injury to an individual drug may be challenging. Given that the vast majority of patients with ChILI are managed with corticosteroids,^9 12^ tools that would allow better stratification of patients and potentially avoiding steroid administration altogether have already shown some promise.^11^

Therefore, there is a need to study the immune characteristics of ChILI in a prospective cohort of patients receiving CPI alongside a well-phenotyped cohort of patients with DILI (due to other medications) and acute presentation of AIH. We aimed to identify immune cell subset signatures of ChILI in conjunction with corroborative cytokine profiles, compared with DILI, AIH, and controls pre-immunotherapy and post-immunotherapy. Using mass cytometry, followed by flow cytometry, single-cell RNA sequencing, liver tissue staining, liver tissue RNA sequencing (RNA-seq), and cytokine profiling, we characterize the immune response during ChILI compared with DILI and AIH.

## Participants and methods

### Patient population and controls

**Figure 1:**
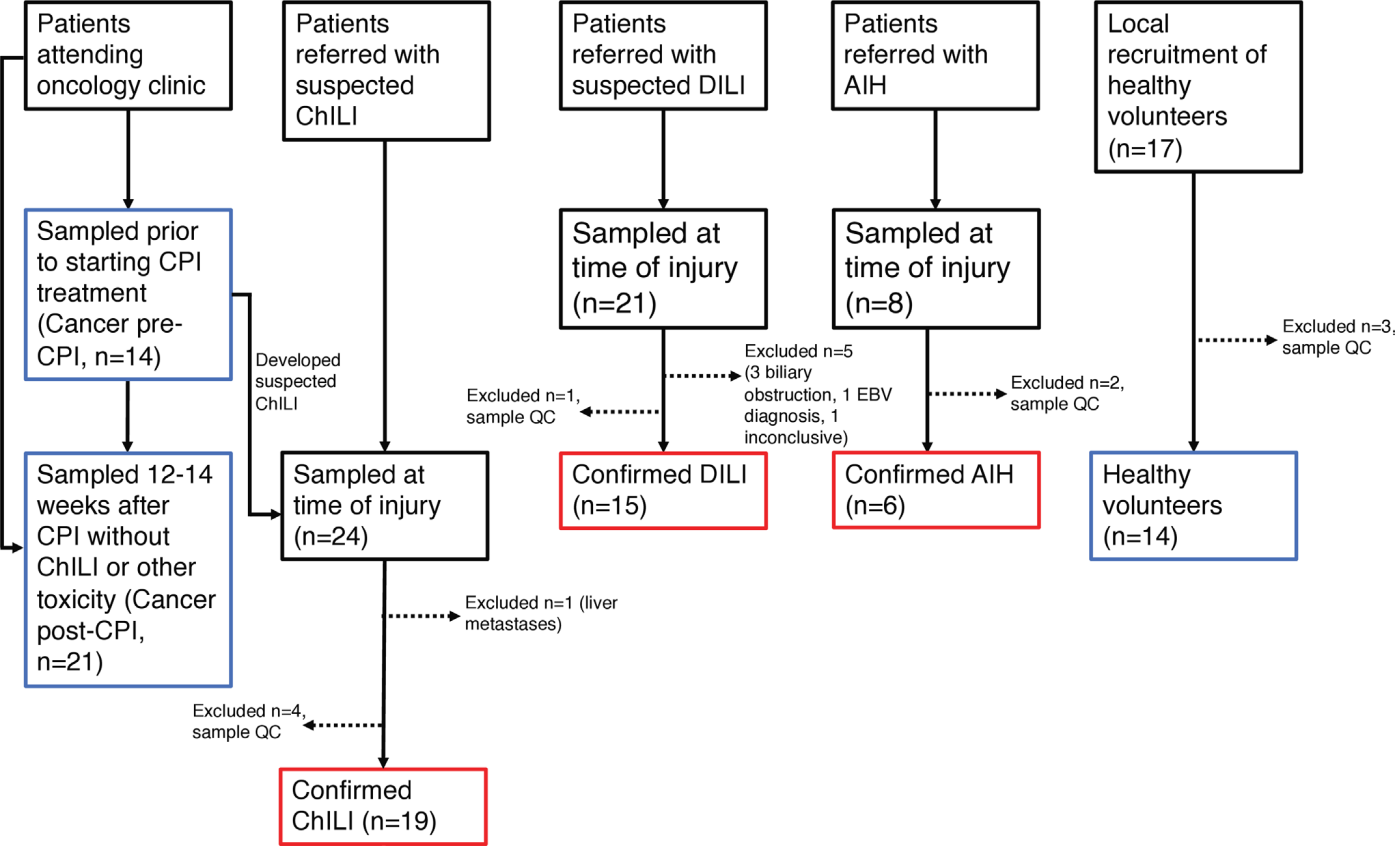
Study flow diagram. AIH, autoimmune hepatitis; ChILI, checkpoint inhibitor-induced liver injury; CPI, checkpoint inhibitors; DILI, drug-induced liver injury; EBV, Epstein-Barr virus; QC; quality control.

The study recruitment pathway is summarized in figure 1. Patients with cancer where CPI alone was indicated as the main therapy were prospectively recruited (trial registration: https://clinicaltrials.gov/study/NCT04476563) at Nottingham University Hospitals NHS trust (NUH). For cancer controls, patients were sampled prior to CPI therapy (Cancer pre-CPI), and after 12 weeks of CPI if they did not develop ChILI or any other irAE (Cancer post-CPI). Patients with ChILI were identified in secondary care through monitoring of treatment with CPI, and patients with DILI and AIH were referred with acute liver injury, with all groups meeting the biochemical criteria described previously.^13^ All were sampled at the time of liver injury, and patients with ChILI were sampled before steroid administration where possible. Healthy adults with no liver disease or cancer diagnosis were enrolled at NUH or University Hospitals Birmingham NHS Trust. Samples from a previous study^11^ of patients with steroid-naïve ChILI, DILI and AIH were obtained for bulk liver tissue RNA-seq and validatory flow cytometry experiments.

### Mass cytometry

Fresh whole blood samples were stained using the Maxpar Direct Immune Profiling Kit (Standard BioTools, (online supplemental table 2). Data were acquired using a Helios mass cytometer (Standard BioTools). Following initial unsupervised clustering using FlowSOM (online supplemental figure 1), analysis was carried out using the R packages CATALYST and diffcyt. CD8^+^ clusters were extracted from the dataset for pseudotime analysis using the R package Slingshot. Cluster annotation is described in detail in online supplemental methods.

### Flow cytometry

Peripheral blood mononuclear cells (PBMC) isolated from whole blood were used for surface and intracellular cytokine staining. Cells were stained with fluorophore-labeled antibodies and matched isotype controls (online supplemental table 3) and data were acquired on an ID7000 spectral flow cytometer (Sony Biotechnology). Data were analyzed in FlowJo V.10 software. Fluorescence minus one controls were used for gating (online supplemental figures 2A and B).

### Single-cell RNA sequencing

PBMC samples from a subset of immunotherapy patients (Cancer pre-CPI, Cancer post-CPI, ChILI, and ChILI follow-up (sampled 4 weeks post-ChILI), 10 per group) were selected for single-cell RNA sequencing (scRNA-seq). scRNA-seq was carried out using the 10x Genomics 5′ Gene Expression and V(D)J library preparation kits. Sequence reads were processed using CellRanger (10x Genomics) and unsupervised clustering was carried out in the R package Seurat using the default Louvain algorithm. Cell clusters were initially annotated using the human PBMC reference (HuBMAP Consortium, (online supplemental figure 3),^14^ before extracting cells annotated as CD8^+^ for re-clustering. Clusters were compared using the R package speckle, using logit transformed proportions. Upstream transcription factors of each cluster were predicted using the package Binding Analysis for Regulation of Transcription (BART).

### Immunofluorescence

Formalin-fixed paraffin-embedded (FFPE) liver tissue was obtained from five ChILI, five DILI and five AIH biopsies. Staining was carried out as described in^15^ using a five-color antibody panel for e-Cadherin, CD8, CD38 and CXCR3 (online supplemental table 4). Cells were counted in QuPath (V.0.5.1) using four randomly selected regions of interest per biopsy at ×10 magnification, with a total area of 2–4 mm^2^ tissue studied per biopsy.

### Bulk RNA sequencing of liver tissue

Snap-frozen liver biopsies from steroid-naïve patients with ChILI, DILI and AIH were obtained from a previously described study.^11^ Clinical data are included in online supplemental table 1. Bulk RNA-seq data from healthy liver tissue was sourced from publicly available studies (NCBI BioProject IDs PRJNA523510 and PRJNA542148). Gene expression fold changes and Gene Set Enrichment Analysis were calculated using DESeq2 and clusterProfiler. The top enriched pathways/Gene Ontology (GO) terms are presented for each condition, relative to healthy liver tissue. Abundance of non-parenchymal cells in liver tissue was estimated using CIBERSORTx,^16^ with a publically available signature matrix generated from scRNA-seq of liver tissue.^17^

### Cytokine profiling

Plasma samples were analyzed via Luminex using the Inflammation 20-plex and Immuno-Oncology 14-plex Human ProcartaPlex panels (Invitrogen). The inflammation panel was used for all sample groups, the immuno-oncology panel was used only for patients with Cancer pre-CPI and post-CPI and ChILI. Patients recruited at baseline who subsequently developed ChILI were separated from the Cancer pre-CPI group to assess the predictive characteristics of inflammatory cytokines (ChILI pre-CPI). As multiple plates were used batch correction was carried out using the R package batchtma.

A detailed description of all methods and software packages used is available in supplementary material.

## Results

### Patient characteristics

40 patients with acute liver injury and 55 controls were prospectively enrolled. Patients with acute liver injury were adjudicated as ChILI (n=19), DILI (n=15) or AIH (n=6). Recruitment and reasons for exclusion from all groups are summarized in figure 1, and clinical characteristics of all groups are summarized in table 1. There was no significant difference in age and liver enzymes at the time of sampling between the three liver injury groups (table 1). Given the female preponderance in AIH and DILI and the male preponderance in metastatic malignant melanoma (the largest tumor type in the ChILI group, 11/19), it was unsurprising to see a significant difference in sex between groups (p=0.007) with females predominant in AIH (5/6) and DILI (12/15) groups compared with ChILI (6/19). Comparison of overall (OS) and progression-free survival (PFS) between patients with ChILI and CPI controls (ChILI n=11, No ChILI n=9, restricted to patients with metastatic melanoma on combination ipilimumab/nivolumab CPI therapy) demonstrated a numerically superior survival in the ChILI group, but this was not statistically significant when tested via Cox proportional hazards model (online supplemental figure 4).

**Table 1:** Clinical characteristics and liver enzymes of patients with acute liver injury at the time of sampling

	ChILI (n=19)	DILI (n=15)	AIH (n=6)	P value*	Control (n=14)	Cancer pre-CPI (n=14)	Cancer post-CPI (n=21)
Mean age, years (SD)	60 (14)	65 (19)	65 (12)	0.62	49 (15)	63 (17)	64 (17)
Sex, n (Male, %)	13 (68.4)	3 (20)	1 (16.6)	0.007	9 (45)	11 (79)	17 (81)
Type of cancer	Malignant melanoma (n=11) Renal cell carcinoma (n=4) Non-small cell lung cancer (n=3) Head and neck (n=1)	NA	NA	NA	NA	Malignant melanoma (n=13) Head and neck (n=1)	Malignant melanoma (n=14) Renal cell carcinoma (n=5) Metastatic Merkel cell carcinoma (n=1) Head and neck (n=1)
CPI regime/class of causative drug	Combination ipilimumab and nivolumab (n=14) Pembrolizumab (n=4) Avelumab (n=1)	Antimicrobial (n=5) Statin (n=4) Anti-TNF (n=2) Chemotherapy (n=2) Anti-TB (n=1) Methotrexate (n=1)	NA	NA	NA	Combination ipilimumab and nivolumab (n=7) Pembrolizumab (n=6) Nivolumab monotherapy (n=1)	Combination ipilimumab and nivolumab (n=7) Pembrolizumab (n=12) Nivolumab monotherapy (n=1) Avelumab (n=1)
Days between start of CPI therapy and sampling Median (Q1, Q3)	46 (37.5, 78)	NA		0.64	NA	NA	84 (42, 89)
Cycles of CPI therapy at time of sampling Median (Q1, Q3)	2 (1, 3)	NA	NA	0.31	NA		2 (2, 3)
Patients received corticosteroids prior to sampling, n (%)	13 (68.4)	0	1 (16.6)	NA	NA	NA	NA
Injury pattern Type† (n, %)	Cholestatic (n=4, 21.1%) Hepatocellular (n=10, 52.6%) Mixed (n=5, 26.3%)	Cholestatic (n=1, 6.7%) Hepatocellular (n=13, 86.7%) Mixed (n=1, 6.7%)	NA	NA	NA	NA	NA
ALT, IU (ULN: 45 males, 35 females) Median (Q1, Q3)	302 (232, 610)	485 (316, 687)	250 (207, 524)	0.5	NA	NA	NA
ALP, IU (ULN: 130) Median (Q1, Q3)	191 (117, 650)	325 (169, 521)	173 (169, 251)	0.41	NA	NA	NA
Total bilirubin, µmol/L (ULN: 21) Median (Q1, Q3)	17 (11, 23)	35 (10, 245)	94 (28, 292)	0.13	NA	NA	NA

* P value was calculated comparing liver injury groups, based on analysis of variance (age), Pearson’s χ2 test (sex) and Kruskal-Wallis rank sum test (ALT, ALP and total bilirubin). ChILI and Cancer post-CPI groups were compared using Mann-Whitney U test (days between start of CPI therapy and sampling, and cycles of CPI therapy at time of sampling).

† Liver injury pattern was defined using the R value calculated as (ALT/ULN)/(ALP/ULN), with R≥5 defined as hepatocellular injury, R≤2 defined as cholestatic and R 2–5 defined as mixed.

AIH, autoimmune hepatitis; ALP, alkaline phosphatase; ALT, alanine transaminase; ChILI, checkpoint inhibitor-induced liver injury; CPI, checkpoint inhibitors; DILI, drug-induced liver injury; IU, international unit; NA, not applicable; TB, tuberculosis; TNF, tumor necrosis factor; ULN, upper limit of normal.

### CPI-induced liver injury results in a significant increase in abundance of a CD8^+^ effector memory population expressing CD38, HLA-DR and CXCR3 along with changes in monocyte ratios and an increase in CD4^+^ regulatory T cells

**Figure 2:**
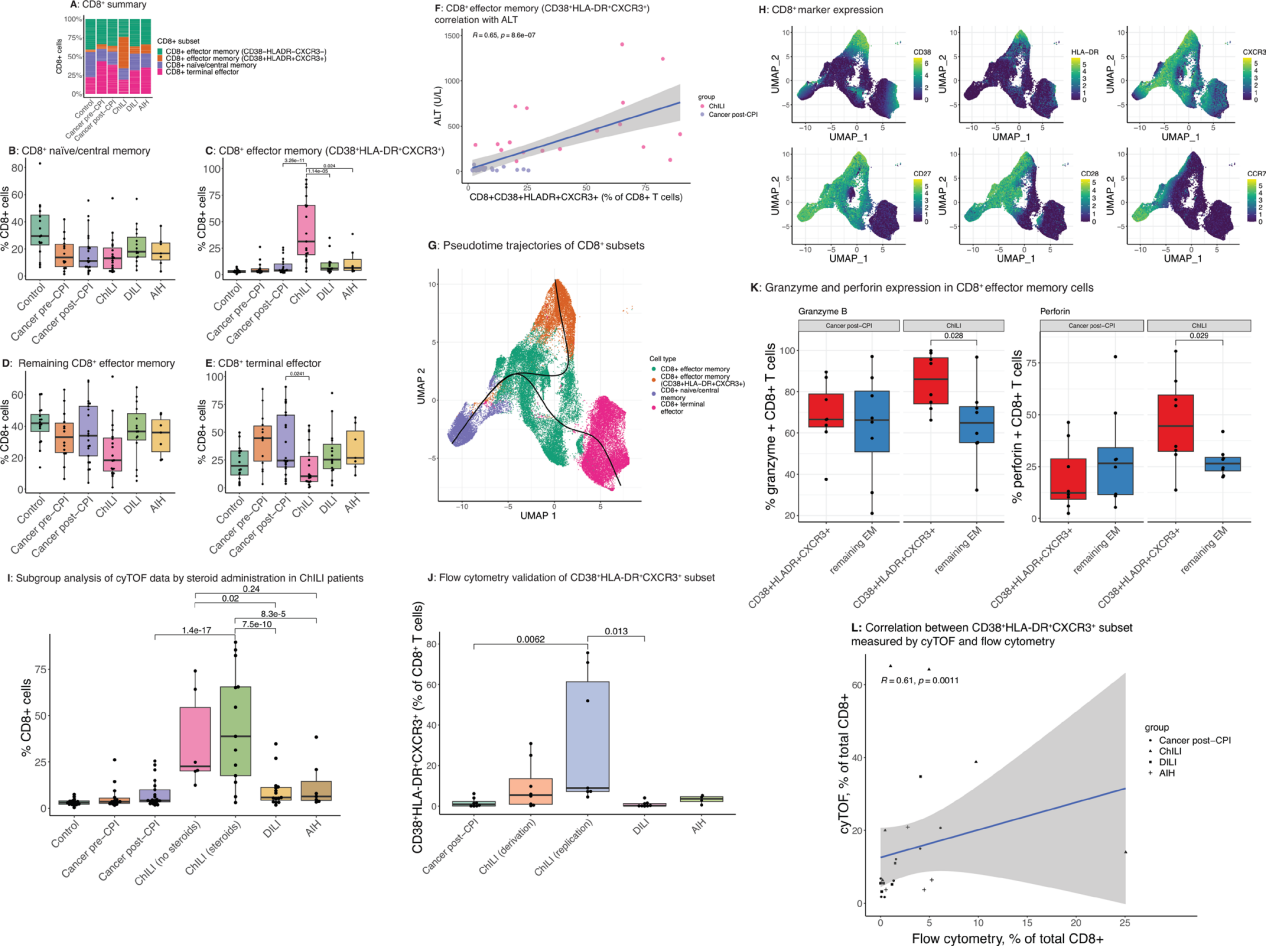
Peripheral CD8^+^ T cells characterized by mass cytometry. Control n=14, Cancer pre-CPI n=14, Cancer post-CPI n=21, ChILI n=19, DILI n=15, AIH n=6. (A) Average percentage abundance of each CD8^+^ cluster by experimental group. (B–E) Abundance of each CD8^+^ cluster as a percentage of total CD8^+^ cells. Differential abundance for graphs B–E was assessed by pairwise comparisons with the diffcyt R package, with all p values adjusted for multiple comparisons using the Benjamini-Hochberg method. (F) Spearman correlation between CD8^+^CD38^+^HLA-DR^+^CXCR3^+^ subset abundance and ALT in patients receiving CPI. (G) UMAP plot, colored by cell cluster with pseudotime trajectories calculated using expression of CD27, CD28, CD45RA, CD45RO, CCR7, CD38, HLA-DR and CXCR3 in the R package Slingshot. (H) UMAP plots colored by expression of CD38, HLA-DR, CXCR3, CD27, CD28 and CCR7. (I) CD8^+^CD38^+^HLA-DR^+^CXCR3^+^ cluster analysis grouping patients with ChILI by whether steroids were administered before sampling. Control n=14, Cancer pre-CPI n=14, Cancer post-CPI n=21, ChILI (no steroids) n=6, ChILI (steroids) n=13, DILI n=15, AIH n=6. Statistical comparisons were made using the diffcyt package in R with p values adjusted using the Benjamini-Hochberg method. (J) CD8^+^CD38^+^HLA-DR^+^CXCR3^+^ subset measured by flow cytometry (Cancer post-CPI n=8, ChILI (derivation) n=8, ChILI (validation) n=7, DILI n=8, AIH n=4). Derivation samples were taken from the original cohort, validation samples were taken from the Barcelona cohort as described in the methods. CD8^+^ cells were defined as CD3^+^CD8^+^ and effector memory cells were gated based on CCR7 and CD45RA expression. (K) Flow cytometry comparison of granzyme B and perforin expression in CD8^+^CD38^+^HLA-DR^+^CXCR3^+^ cells and remaining effector memory cells in ChILI and cancer post-CPI controls. (L) Spearman correlation between mass and flow cytometry where matched samples were available (Cancer post-CPI n=6, ChILI n=6, DILI n=6, AIH n=4). AIH, autoimmune hepatitis; ALT, alanine transaminase; ChILI, checkpoint inhibitor-induced liver injury; CPI, checkpoint inhibitors; cyTOF, cytometry by time-of-flight; DILI, drug-induced liver injury; UMAP, Uniform Manifold Approximation and Projection.

Mass cytometry revealed a significant increase in a peripheral circulating CD8^+^ effector memory cell subset in patients with ChILI in comparison to healthy, patients with cancer pre-CPI and post-CPI, and in comparison to DILI and AIH (figure 2A and C). Concurrently, the terminal effector CD8^+^ subset was significantly reduced in patients with ChILI relative to Cancer post-CPI controls (figure 2A and E). The remaining CD8^+^ clusters were not significantly changed between any groups (figure 2B and D). The abundance of the CD8^+^CD38^+^HLA-DR^+^CXCR3^+^ subset was significantly correlated with alanine transaminase (ALT) in patients receiving CPI (figure 2F). Pseudotime analysis of this subset demonstrated a distinct bifurcation of the CD38^+^HLA-DR^+^CXCR3^+^ subpopulation away from the remaining CD8^+^ effector memory cells (figure 2G and H). Subgroup analysis dividing the ChILI group into patients sampled pre-steroid and post-steroid administration showed steroids had no significant effect on this subset (figure 2I).

Flow cytometry of PBMC taken from a subset of patients used for mass cytometry, and PBMC taken from a separate cohort of steroid-naïve patients validated the mass cytometry findings (figure 2J). Intracellular cytokine staining revealed CD38^+^HLA-DR^+^CXCR3^+^ cells express more granzyme B and perforin compared with other CD8^+^ effector memory cells, and this is more pronounced in ChILI (figure 2K). In matched whole blood and PBMC samples the CD38^+^HLA-DR^+^CXCR3^+^ subset measured by flow cytometry was significantly correlated with the same subset measured by mass cytometry (figure 2L, R=0.61, p=0.0011).

Of the remaining cell subsets analyzed, we observed a significant increase in CD4^+^ regulatory T cells in ChILI relative to Cancer post-CPI controls and DILI (online supplemental figure 5). Monocyte ratios were also significantly altered in ChILI, with a significant increase in classical monocytes with a concurrent reduction in intermediate and non-classical monocytes (online supplemental figure 6).

### Single-cell RNA sequencing in matched samples corroborates mass cytometry results and indicates an exhausted phenotype characteristic of chronic antigen stimulation

**Figure 3:**
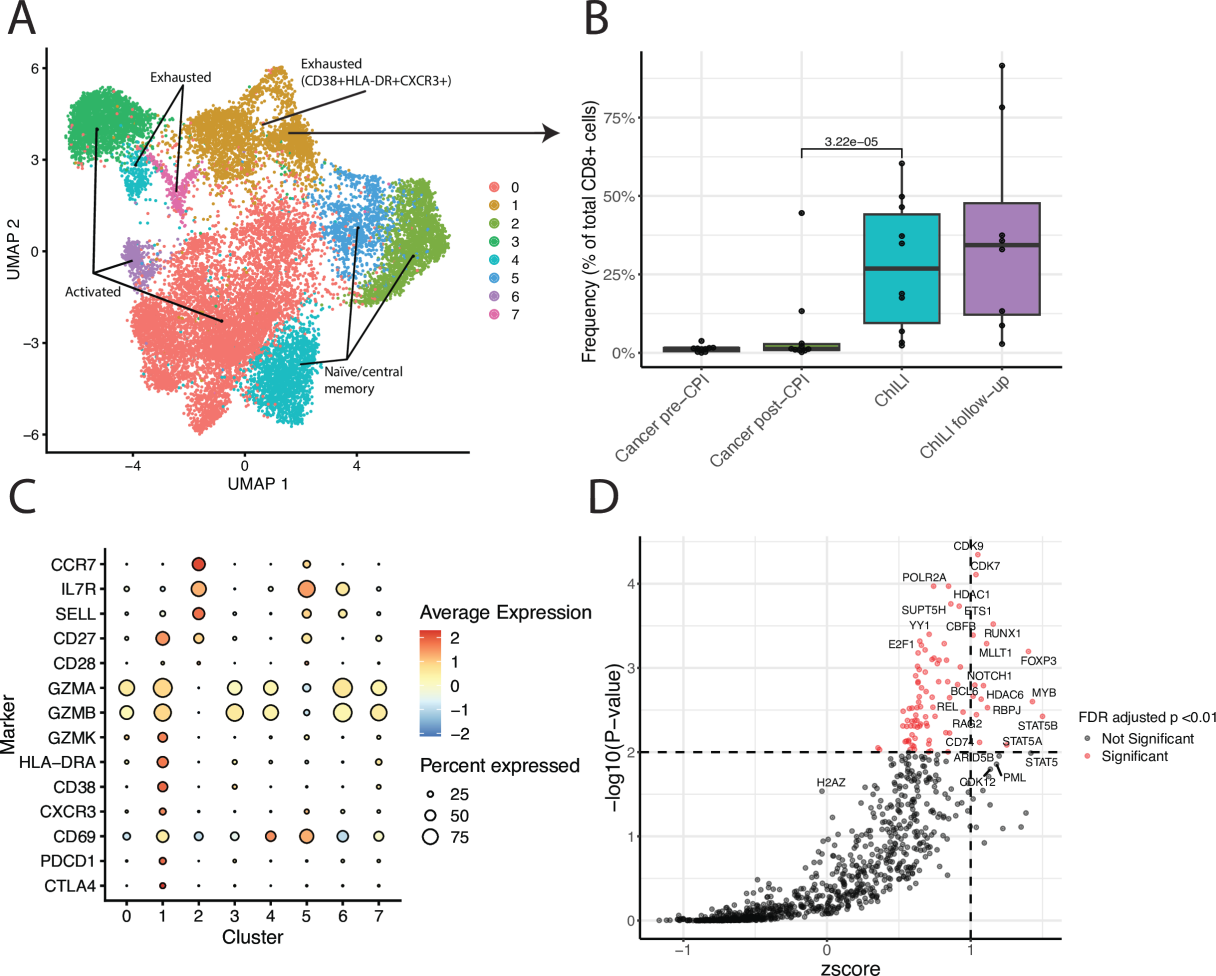
Single-cell RNA sequencing of Cancer pre-CPI (n=10), cancer post-CPI (n=10), ChILI (n=10), and patients sampled 4 weeks post-ChILI (ChILI follow-up, n=10). (A) UMAP plot of CD8^+^ clusters assigned in Seurat using a Louvain algorithm. (B) Relative abundance of cluster 1 (CD8^+^CD38^+^HLA-DR^+^CXCR3^+^) as a percentage of total CD8^+^ cells, statistical comparisons are made using logit transformed cluster proportions and a regression model fitted using the R package limma. (C) Mean expression of selected markers across all clusters. (D) Prediction of key upstream transcription factors mediating gene expression in the CD8^+^CD38^+^HLA-DR^+^CXCR3^+^ cluster, key genes were determined using the FindMarkers function in Seurat, filtered to those with FDR adjusted p<0.05, the resulting gene list was used as the input for the Binding Analysis for Regulation of Transcription package. Putative transcription factors were filtered based on z-score >1 and Irwin-Hall corrected p value<0.01. ChILI, checkpoint inhibitor-induced liver injury; CPI, checkpoint inhibitors; FDR, false discovery rate; UMAP, Uniform Manifold Approximation and Projection.

Following clustering and cell type identification (online supplemental figure 4), CD8^+^ cells were isolated and re-clustered (figure 3A). A cluster (cluster 1) was identified corroborating the protein expression work, present only in patients with ChILI sampled in the acute phase of injury and persisting at follow-up (4 weeks after acute sample). Differential abundance testing confirmed a significant elevation of this cluster in patients with ChILI (figure 3B). These cells expressed significant amounts of CD38, HLA-DR, and CXCR3, as well as GZMK, exhaustion markers CTLA-4, PDCD1 (coding for PD-1), HAVCR2, and the liver residency marker CD69 (figure 3C). Prediction of transcription factors using the Binding Analysis for Regulation of Transcription package (BART, V.2.0), using markers significantly enriched in the cluster associated with ChILI, suggested that these cells are chiefly regulated by CDK9, CDK7, RUNX1 and FOXP3 (figure 3D).

### Tissue immunofluorescence and transcriptomic analyses reveal enrichment of liver-resident CD8^+^ T cells and CXCR-related pathways in ChILI versus DILI and AIH, and presence of CD8^+^CD38^+^CXCR3^+^ cells in the liver

**Figure 4:**
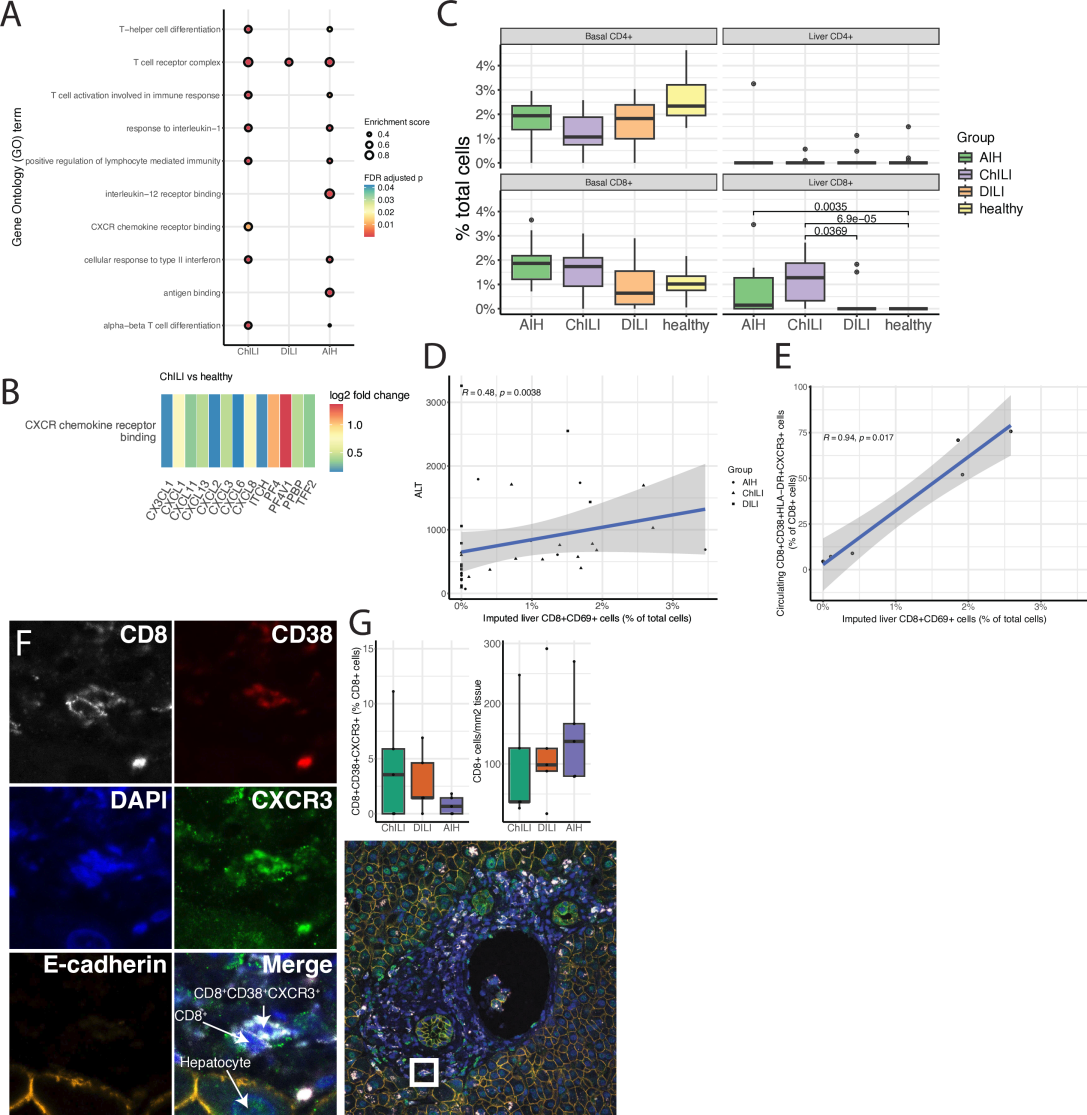
Bulk RNA-seq results from snap-frozen liver biopsies in ChILI (n=16), DILI (n=10) and AIH (n=10) compared with data from publically available healthy liver tissue (n=16). (A) Top 10 enriched GO terms in the three experimental groups relative to healthy liver tissue. (B) Heatmap showing log2 fold change between ChILI and healthy liver tissue of genes contained within the GO term “CXCR chemokine receptor binding”. (C) Abundance of immune cell populations in liver tissue estimated from bulk RNA-seq data using CIBERSORTx in ChILI (n=16), DILI (n=10), AIH (n=10) and healthy liver tissue (publically available data, n=16). Significance was tested between groups via Mann-Whitney U test. (D) Spearman correlation in all patient groups between imputed liver CD8^+^CD69^+^ cells and ALT. (E) Spearman correlation in patients with ChILI between peripheral CD8^+^CD38^+^HLA-DR^+^CXCR3^+^ T-cells measured by flow cytometry, and resident (CD69^+^) CD8^+^ T cells imputed from bulk tissue RNA-seq data (where matched blood and biopsy tissue was available, n=6). (F) Immunofluorescence staining of CD38^+^CXCR3^+^ CD8^+^ T cells in ChILI, with representative example of CD8+infiltration in ChILI. (G) (Left) Comparison of CD8^+^CD38^+^CXCR3^+^ cells in ChILI (n=5), DILI (n=5) and AIH (n=5) biopsies, expressed as a percentage of total CD8^+^ cells. (Right) Total numbers of CD8^+^ cells in each biopsy expressed as cells per mm^2^ of tissue. AIH, autoimmune hepatitis; ALT, alanine transaminase; ChILI, checkpoint inhibitor-induced liver injury; CPI, checkpoint inhibitors; DILI, drug-induced liver injury; GO, Gene Ontology; RNA-seq, RNA sequencing.

Gene set enrichment using GO terms demonstrated significant enrichment of gene sets relating to T-cell differentiation and activation in ChILI relative to AIH and DILI, with more similarities observed between ChILI and AIH (figure 4A). The GO term CXCR chemokine receptor binding was significantly enriched in ChILI biopsies relative to other groups, all genes within this GO term were upregulated in ChILI relative to healthy liver tissue, with CXCL1, CXCL8 and PF4 (CXCL4) showing the greatest change (figure 4B). Aside from the broad GO term “T-cell receptor complex” (205 genes), no GO term was enriched in DILI relative to healthy liver tissue.

Imputation of immune cell fractions from liver bulk RNA-seq data using CIBERSORTx revealed a significant increase in resident CD8^+^ cells (defined as CD8^+^CD69^+^) in ChILI (n=16) biopsies relative to those from patients with DILI (n=10) and AIH (n=10) (figure 4C). Across all patient groups, the imputed CD8^+^CD69^+^ liver resident population was significantly correlated with ALT (R=0.48, p=0.0038, figure 4D). Where matched PBMC and tissue samples were available in patients with ChILI, imputed liver resident CD8^+^CD69^+^ cells were significantly correlated with circulating CD8^+^CD38^+^HLA-DR^+^CXCR3^+^ cells measured by flow cytometry (R=0.94, p=0.017, figure 4E). No differences were observed in other immune cell types with either a basal or liver resident phenotype.

Using immunofluorescence (figure 4F), the proportion of CD8^+^CD38^+^CXCR3^+^ T cells in liver biopsy tissue was calculated and compared between ChILI (n=5), DILI (n=5) and AIH (n=5) samples. CD8^+^CD38^+^CXCR3^+^ cells were present in all conditions in small numbers, with no significant differences observed between conditions (figure 4G).

### Inflammatory cytokines show some commonalities between ChILI and AIH, while sPD-1 and sCD27 are indicative of ChILI

**Figure 5:**
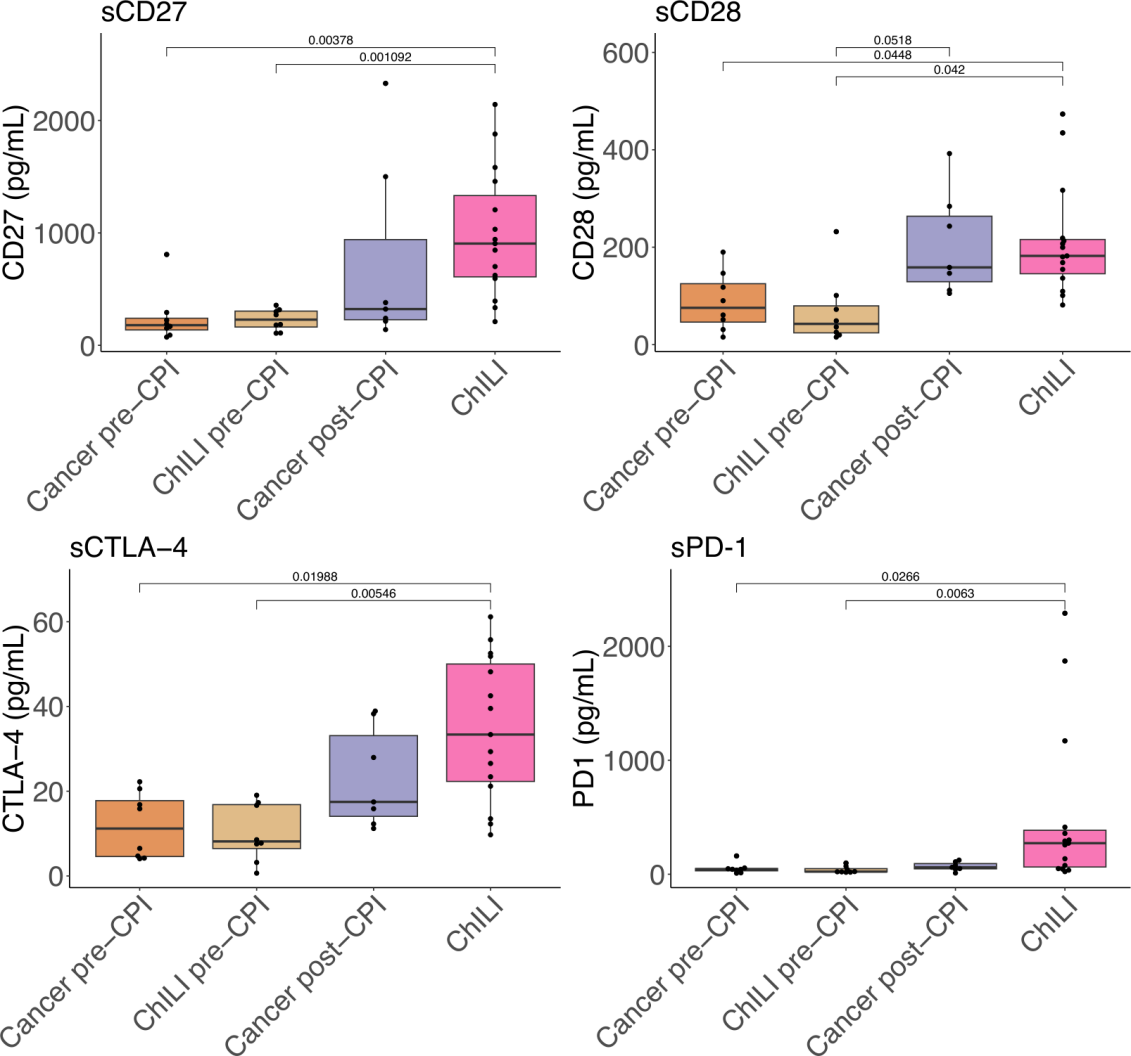
Plasma cytokines in a subset of ChILI cases and controls. Where available, pre-CPI samples were split into those who did and did not subsequently develop ChILI. Cancer pre-CPI n=8, ChILI pre-CPI n=8, Cancer post-CPI n=7, ChILI n=15. All statistical comparisons are by Mann-Whitney U test. ChILI, checkpoint inhibitor-induced liver injury; CPI, checkpoint inhibitors; CTLA-4, cytotoxic T-lymphocyte-associated protein 4; PD-1, programmed cell death protein 1; sCTLA-4, soluble CTLA-4; sPD-1; soluble PD-1.

A number of inflammatory cytokines were significantly elevated in the three liver injury groups, with the majority of these (interleukin (IL)-12, IL-13, IL-1α, IL-1β, IL-4, IL-8, C-C motif chemokine ligand 4 (CCL4)) only significantly increased in AIH (online supplemental figure 7). IL-10, CCL3 and CXCL10 were significantly elevated in both ChILI and AIH, relative to DILI and other control groups (online supplemental figure 7). On accounting for steroid administration, CCL3 was no longer significantly elevated in ChILI, but IL-10 and CXCL10 remained unchanged (online supplemental figure 8). In patients with ChILI recruited pre-CPI therapy (ChILI pre-CPI), no inflammatory cytokines demonstrated any predictive effects. Soluble PD-1 and soluble CTLA-4 were both significantly increased in ChILI cases relative to pretreatment groups (figure 5). sCTLA-4 was also elevated in post-CPI controls but not significantly, whereas sPD-1 was only elevated in the ChILI group. Circulating levels of soluble CD27 (sCD27) were significantly elevated in ChILI cases relative to pretreatment groups, and sCD28 was significantly elevated in both ChILI and post-CPI controls. Again, no predictive effects were observed in the ChILI pre-CPI group.

## Discussion

We have identified a population of exhausted CD8^+^ effector memory cells in the peripheral circulation as the most dominant CD8^+^ T-cell subset in ChILI. This CD8^+^ subtype differentiates patients with ChILI from patients receiving CPI with no other irAE, patients with DILI due to other drugs, and AIH. In patients receiving CPI, these cells were significantly correlated with the degree of liver damage as reflected by the serum concentration of ALT. Concurrently, peripheral CD8^+^ terminal effector cells were significantly lower in patients with ChILI. Pseudotime modeling showed that this subset formed its own divergent trajectory away from the remaining effector memory population, suggesting differing patterns of T-cell specificity.^18^

The presence of this subset in patients with ChILI samples was validated by scRNA-seq and flow cytometry, which both demonstrated the increased cytotoxic potential of this population relative to other CD8^+^ cells. Flow cytometry also demonstrated upregulation of this subset in steroid-naïve patients with ChILI from a separate cohort. This highlights the fact that these cells can be identified readily by flow cytometry, and this CD8^+^ subset is apparent regardless of steroid administration (also demonstrated by subgrouping the derivation cohort, figure 2I). Immunofluorescence showed the presence of these cells in FFPE biopsies, again despite steroid administration, and tissue bulk RNA-seq confirmed upregulation of T-cell response pathways, specifically CXCR chemokine receptor response, and suggested increased CD8^+^ T-cell liver residency in ChILI.

For the first time, we have characterized this CD8^+^ subtype in ChILI, but peripheral indicators of CD8^+^ activation have been observed previously.^10^ In a related study, where all patients with ChILI were managed using a two-step algorithm including discontinuation of CPI first followed by assessment of the degree of inflammation using liver biopsy, total HLA-DR expression on peripheral blood CD8^+^ cells positively correlated with the degree of liver inflammation on histology and progression of ChILI requiring corticosteroid treatment.^11^ There is growing concern that patients receiving early, high-dose corticosteroids for irAEs have poorer OS and PFS after the onset of the adverse event.^19 20^ Therefore, there is an opportunity to evaluate the utility of CD38^+^HLA-DR^+^CXCR3^+^ CD8^+^ cells as a marker to stratify patients who may require immunosuppression for the treatment of ChILI.

CD38 plays a role in T-cell migration and activation and antigen presentation, particularly in autoimmune disorders,^21^ but despite this, its mechanistic role is not clear. Expression of CD31, the cognate antigen for CD38, on liver sinusoidal endothelial cells (LSEC) provides a mechanism for the circulating CD8^+^CD38^+^ subset to adhere and infiltrate into the liver, but expression of CD31 on LSECs is debated.^22^ CD38 also plays several roles in inflammation, by modulating expression of IL-1β and tumor necrosis factor-α, and by depletion of cellular nicotinamide adenine dinucleotide (NAD+).^21^ Similar CD38^+^ T-cell populations have been identified in the joints and blood of patients with CPI-induced arthritis.^23^

While the mass cytometry data suggests this subset is effector memory due to a lack of re-expression of CD45RA, the increased resolution of the scRNA-seq data shows high expression of the exhaustion markers PDCD1, CTLA-4 and HAVCR2, suggesting prolonged antigen stimulation and an exhausted phenotype. CD38 expression is also sustained in these exhausted cells, with CD38 shown to be a regulator of exhausted CD8^+^ T cells in chronic viral infections^24^ and cancer.^25^ scRNA-seq also revealed a significant increase in GZMK relative to other subsets, and the tissue residency marker CD69. Prediction of significant transcription factors using the marker genes for the CD38^+^HLA-DR^+^CXCR3^+^ cluster revealed a number of links to autoimmunity, chiefly the cyclin-dependent kinases 9 and 7,^26 27^ and RUNX1.^28^ These have been demonstrated recently as regulators of auto-reactive cells in non-alcoholic steatohepatitis.^29^

FFPE biopsies were available from ChILI, DILI and AIH cases for immunofluorescent staining. We identified CD8^+^CD38^+^CXCR3^+^ T cells in all groups, but only in small numbers relative to total CD8^+^ cells. At the time of liver biopsy, patients with ChILI were on corticosteroid treatment which could have dampened the inflammatory cell infiltration,^30^ whereas neither patients with DILI nor AIH received corticosteroids. Shojaie *et al* have suggested it is links between CD8^+^ T cells, macrophages and apoptotic cleaved caspase 3+ hepatocytes causing ChILI, using imaging mass cytometry in a murine model,^31^ therefore applying similar higher resolution techniques to human biopsies would be worthwhile.

Although drawn from a different cohort to the peripheral blood and FFPE biopsy samples, the samples used for tissue RNA-seq were broadly similar in terms of cancer and CPI treatments (online supplemental table 1). We used these as liver tissue was obtained from steroid-naïve patients with ChILI. These revealed upregulation of several GO gene sets related to T-cell differentiation and antigen binding, the majority of which were similar between ChILI and AIH groups indicating some overlap between these conditions as highlighted previously.^32^ Most interestingly, the gene set related to CXCR chemokine receptor binding was significantly enriched in ChILI relative to other groups, providing further evidence for CXCR3^+^ CD8^+^ T cells infiltrating the liver. A number of upregulated genes in this set, including PF4 (CXCL4) and CXCL11 will bind to CXCR3.^33^ Additionally, imputation using CIBERSORTx showed a significant increase in liver resident CD8^+^ cells (defined as CD8 cells with high CD69 expression) in ChILI biopsies. Tissue resident T cells are known to play a role in response to CTLA-4 and PD-1 immunotherapies,^18^ as a pre-existing reservoir which then proliferates in response to CPI treatment,^34^ similar reservoirs exist in chronic viral infections and also rapidly proliferate in response to CPI.^35^ Although only available in a small subset of samples (n=6) with matched PBMC and biopsy, circulating CD8^+^CD38^+^HLA-DR^+^CXCR3^+^ were significantly correlated with imputed liver-resident CD8^+^CD69^+^ cells, as well as ALT. This suggests that the CD8^+^ T-cell population identified in this study plays a major role in liver damage in ChILI, relative to AIH and DILI, but it remains unclear whether it originated from a pre-existing liver-resident population. Recent work linking cytomegalovirus seropositivity with improved survival following anti-PD-1 checkpoint therapy, as well as reduced toxicity (although not including ChILI) suggests previous immune exposure warrants further exploration.^36^

There are a number of mouse models developed to study DILI and ChILI, these involve either blocking CTLA-4 and PD-1 directly,^37^ or knocking out PD-1 before administering anti-CTLA-4 antibodies plus a third factor to induce toxicity, such as indoleamine-2,3-dioxygenase inhibitor to promote T-cell activation,^38 39^ amodiaquine^40^ or TLR9.^41^ These models have demonstrated liver infiltration of CD8^+^ cells in ChILI,^38 39^ with upregulation of CXCR3, granzyme and perforin expression also noted in this infiltrate. Previous work in both CD4^+^ and CD8^+^ T cells has also shown that CXCR3 is essential for migration across hepatic sinusoids,^42 43^ and it has been linked to T-cell infiltration in a number of other conditions, including other irAEs.^44^ Blockade of CCR2 using either a knockout model (Ccr2^rfp/rfp^) or a CCR2 agonist (Cenicriviroc), preventing monocyte/CD8^+^ interaction appears to ameliorate ChILI.^41^ Replicating previous work,^10^ we observed a similar shift in monocyte ratios in ChILI, with a significant increase in classical monocytes (CD14^high^CD16^−^), suggesting monocyte recruitment to the liver, and again suggesting monocyte/CD8^+^ T-cell crosstalk is central to liver inflammation in ChILI.

Similarly to the PD-1^−/−^/amodiaquine animal model,^40^ we noted a small but statistically significant increase in total CD4^+^ regulatory T cells in patients with ChILI relative to post-CPI controls (online supplemental figure 6). Further work is necessary to phenotype these cells and clarify if this failure of the T^Reg^ population to adequately suppress the CD8^+^ mediated liver inflammation is simply a result of lack of numbers, or defective T^Reg^ cells, with both implicated in a number of autoimmune diseases.^45^

Of the inflammatory cytokines measured, only IL-10 and CXCL10 stand out as significantly elevated in ChILI. In the context of CD8^+^ T-cell exhaustion IL-10 upregulation from antigen-presenting cells is characteristic of persistent exposure to antigen, and will lead to dysfunctional T-cell responses.^46^ Increased IL-10 secretion is also characteristic of the response to self-antigens in AIH,^47^ as is CXCL10, which mediates T-cell infiltration into various tissues in autoimmunity.^48^ Analysis of cytokines related to immune checkpoint pathways showed an increase in sCD27, sCD28 and sCTLA-4 following CPI administration, regardless of whether patients had ChILI, indicating T-cell activation. Interestingly, sPD-1 was only elevated in patients with ChILI (figure 5). To our knowledge, this has not been demonstrated previously in ChILI, PD-1 blockade primarily affects already activated T cells and therefore results in a narrower mechanism of action compared with CTLA-4 inhibition, which potentially explains the lower frequency of irAEs in single-agent anti-PD-1 therapy compared with combination or anti-CTLA-4.^49^ sPD-1 has been associated with liver inflammation and fibrosis in chronic hepatitis B virus (HBV) infection, along with similar CD8^+^ T-cell exhaustion.^50^

Our study has several strengths; to our knowledge, this is the first study comparing matched immune cell populations and cytokines in three types of immune-mediated liver disease, with strict recruitment and adjudication criteria applied to all cases, and all patients sampled early during the acute phase of liver injury. We have included two substantial control groups of patients pre-CPI and post-CPI without any irAEs to control for changes attributable to the underlying cancer as well as exposure to CPI. In addition, we have demonstrated that flow cytometry is able to identify these specific cell signatures accurately in this patient group. Therefore, it would be feasible to use these cell signatures as a biomarker in a clinical immunology setting to differentiate ChILI from other etiologies that manifest with raised liver enzymes, including DILI due to other medications.

There are limitations to acknowledge. First, patients with ChILI were often treated with steroids before sampling, and this is the case for 63% of the ChILI group in our study. However, subgroup analysis showed that steroid administration to treat ChILI did not have a significant effect on the CD38^+^HLA-DR^+^CXCR3^+^ subset identified, similar to other immunophenotyping work in ChILI.^10^ We also present replication of the flow cytometry data in steroid-naïve patients with ChILI drawn from a separate cohort. In the case of FFPE biopsy samples taken from patients with ChILI, corticosteroids would have influenced the extent of inflammation. We were also limited by a five-color confocal microscopy approach, whereas broader proteomics techniques would allow better resolution of the CD8^+^ subset. As liver biopsies are not routinely taken in cases of ChILI, other avenues such as fine needle aspirates (FNA) could be investigated to study liver resident immune cells in ChILI. FNA have already been proposed as a rapid and safer alternative to biopsies in viral hepatitis to sample intrahepatic immune cells and monitor response to therapy, with good concordance with conventional biopsies,^51^ and have recently been used to study CPI response in hepatocellular carcinoma.^52^

Our cancer post-CPI sample group was restricted to patients with no other irAE, making it impossible to ascertain whether the CD8^+^ subset identified in ChILI is liver specific, or could play a role in other irAE. Limited work in six cancer post-CPI patients with other organ toxicities suggests that this subset is unique to ChILI (online supplemental figure 9), but a larger more well-defined group would be required to show this conclusively.

In conclusion, we have demonstrated an expanded population of peripheral CD38^+^HLA-DR^+^CXCR3^+^ CD8^+^ effector memory cells that are elevated in ChILI but not DILI and AIH. These cells show increased cytotoxic potential relative to other CD8^+^ effector memory cells and are indicative of chronic self-antigen stimulation. CD8^+^ cells expressing both CXCR3 and CD38 are observed in liver tissue as part of CD8^+^ infiltration in ChILI, as well as concomitant signaling pathways using RNA-seq. This work highlights the potential of CD38^+^HLA-DR^+^CXCR3^+^ CD8^+^ T cells as a cell-based biomarker, and suggests some key mechanistic differences between the pathogenesis underlying ChILI, DILI and AIH.

## Supplementary material

10.1136/jitc-2025-014178 online supplemental file 1

10.1136/jitc-2025-014178 online supplemental file 2

10.1136/jitc-2025-014178 online supplemental file 3

## Data Availability

Data are available in a public, open access repository.
