# Supplementary material for: Circulating exhausted CD8+ effector memory cells differentiate immune checkpoint inhibitor-induced liver injury from other acute immune-mediated liver injuries
Source: J Immunother Cancer. 2026 Mar 27;14(3):e014178. doi: 10.1136/jitc-2025-014178 (PMC13034233; doi:10.1136/jitc-2025-014178)
Supplement: online supplemental file 1 [file jitc-14-3-s001.pdf]

Checkpoint Inhibitor-Induced Liver Injury (ChILI) Study

Final version 1.6
26/02/2024

**Short title:**	Checkpoint inhibitor-induced liver injury study
**Acronym:**	ChILI
**IRAS Project ID:**	274603
**Study Sponsor:**	University of Nottingham
**Sponsor reference:**	20034
**REC reference:**	20/NW/0274
**Funding Source:**	Pfizer Inc, University of Nottingham, NIHR Nottingham Biomedical Research Centre

This protocol is confidential and the property of the University of Nottingham. No part of it may be transmitted, reproduced, published, or used by other persons without prior written authorisation from the University of Nottingham

STUDY PERSONNEL AND CONTACT DETAILS

Sponsor: University of Nottingham

Contact name: Ms Angela Shone
Research and Innovation
University of Nottingham
East Atrium
Jubilee Conference Centre
Triumph Road
Nottingham
NG8 1DH

Chief investigator: Professor Guruprasad P Aithal
GI and liver disorders theme lead,
NIHR Nottingham Biomedical Research Centre,
Nottingham University Hospitals NHS Trust and
University of Nottingham, Queens Medical Centre,
Nottingham, NG7 2UH
Phone: 0115 823 1149 (PA)
Email: guru.aithal@nottingham.ac.uk

Co-investigators: Professor Poulam Patel
The Academic Unit of Clinical Oncology
Nottingham City Hospital Campus
Hucknall Road, Nottingham NG5 1PB
Email: poulam.patel@nottingham.ac.uk

Dr Ankit Rao
Consultant medical oncologist
Nottingham City Hospital Campus
Hucknall Road, Nottingham NG5 1PB
Email: Ankit.Rao@nuh.nhs.uk

Dr Arvind Arora
Consultant medical oncologist
Nottingham City Hospital Campus
Hucknall Road, Nottingham NG5 1PB
Email: Arvind.Arora@nuh.nhs.uk

Dr Jason Adhikaree
Consultant medical oncologist
Nottingham City Hospital Campus
Hucknall Road, Nottingham NG5 1PB
Email: Jason.Adhikaree2@nuh.nhs.uk

Dr Arjuna Singanayagam
Consultant hepatologist

This protocol is confidential and the property of the University of Nottingham. No part of it may be transmitted, reproduced, published, or used by other persons without prior written authorisation from the University of Nottingham

St. George's University of London,
Institute of Infection and Immunity
Blackshaw Road, Tooting,
London SW17 0QT
Email: asingana@sgul.ac.uk

Dr Jane Grove
Assistant Professor in Hepatology
NIHR Nottingham Biomedical Research Centre,
Nottingham University Hospitals NHS Trust and
University of Nottingham, Queens Medical Centre,
Derby Road, Nottingham, NG7 2UH
Telephone: 0115 9249924 ext: 64429
Email: jane.grove@nottingham.ac.uk

Dr Stuart Astbury
Scientific research fellow
NIHR Nottingham Biomedical Research Centre,
Nottingham University Hospitals NHS Trust and
University of Nottingham, Queens Medical Centre,
Derby Road, Nottingham, NG7 2UH
Telephone: 0115 9249924 ext: 64429
Email: stuart.astbury@nottingham.ac.uk

Dr Philip Kaye
Consultant histopathologist
Nottingham University Hospitals NHS Trust,
Derby Road, Nottingham, NG7 2UH
Email: philip.kaye@nuh.nhs.uk

Pfizer lead: Changhua Ji (Immuno-safety Science)

Pfizer co-lead/sponsor: Shashi Ramaiah (Safety Biomarkers and Translational Sciences)

Statistician: Dr Dingzhou Li
Associate Director
Pfizer Inc., Eastern Point Road, Groton, CT 06340, USA
Phone: 1-860-7156713
Email: Dingzhou.Li@pfizer.com

Study Coordinator: Elinor Cross
NIHR Nottingham Biomedical Research Centre,
Nottingham University Hospitals NHS Trust and
University of Nottingham, Queens Medical Centre,
Derby Road, Nottingham, NG7 2UH
Email: Elinor.cross@nottingham.ac.uk

This protocol is confidential and the property of the University of Nottingham. No part of it may be transmitted, reproduced, published, or used by other persons without prior written authorisation from the University of Nottingham

Study Coordinating Centre: NIHR Nottingham Biomedical Research Centre, Nottingham University Hospitals NHS Trust and University of Nottingham, Queens Medical Centre, Derby Road, Nottingham, NG7 2UH

STUDY SYNOPSIS

Title	Checkpoint inhibitor-induced liver injury
Acronym	ChILI
Study email	Chili@nottingham.ac.uk
Chief Investigator	Guruprasad P Aithal
Study centres	• Nottingham Digestive Diseases Centre, NIHR Nottingham Biomedical Research Centre, Nottingham University Hospitals NHS Trust and University of Nottingham, Queens Medical Centre, Nottingham NG7 2UH<br>• St. George's University of London, Institute of Infection and Immunity Blackshaw Road, Tooting, London SW17 0QT<br>• Across UK NHS Secondary Care sites (Including Scotland)
Objectives	• To identify the incidence and risk factors for checkpoint inhibitor-induced liver injury (ChILI) and the association with other adverse reactions (especially colitis) by studying a prospective cohort of well characterized patients undergoing checkpoint inhibitor treatment in the real-world setting.<br>• To identify specific phenotypic features associated with checkpoint inhibitor-induced liver injury.<br>• To enrol a similarly well-characterised control group of patients who are treated with checkpoint inhibitors but who do not develop ChILI.<br>• To collect and store biological samples (blood, urine, stool) from patients with ChILI at 3 time points (the day of liver injury, 1 week and 1 month after). We will perform liver biopsy when it is clinically indicated.<br>• To collect and store biological samples (blood, urine and stool) from control patients at a two time points (before starting CPIs and 6 to14 weeks after commencing treatment)<br>• To determine the histological characteristics of the liver in patients with ChILI using available diagnostic samples
Number of participants / tissue samples	250 in total or 120 completed control cases and 40 DILI cases, whichever occurs earlier.<br>Plus anonymised liver tissue from up to 75 biopsies

This protocol is confidential and the property of the University of Nottingham. No part of it may be transmitted, reproduced, published, or used by other persons without prior written authorisation from the University of Nottingham

Diagnosis and main criteria for inclusion	Symptomatic or asymptomatic adults diagnosed with checkpoint inhibitor-induced liver injury OR adults (without symptoms) undergoing checkpoint inhibitor therapy without evidence of ChILI (control group).
Duration of study	5 years
Description of interventions	ChILI patients and controls who are on checkpoint inhibitor treatment: Blood, urine and stool samples will be collected at each visit.<br>For symptomatic patients only: we plan to collect any surplus liver biopsy samples taken as part of clinical care and any archived surplus paraffin sections taken previously as part of clinical care.
Statistical methods	Descriptive statistics

This protocol is confidential and the property of the University of Nottingham. No part of it may be transmitted, reproduced, published, or used by other persons without prior written authorisation from the University of Nottingham

Table of contents

STUDY PERSONNEL AND CONTACT DETAILS 2
Sponsor 2
Chief investigator 2
Co-investigators 2
Statistician 3
STUDY SYNOPSIS 4
ABBREVIATIONS 7
STUDY BACKGROUND INFORMATION AND RATIONALE 8
STUDY OBJECTIVES AND PURPOSE 9
PURPOSE 9
PRIMARY OBJECTIVE 10
SECONDARY OBJECTIVES 10
STUDY DESIGN 10
STUDY CONFIGURATION 10
Primary endpoint 12
Secondary endpoint 12
STUDY MANAGEMENT 12
DURATION OF THE STUDY AND PARTICIPANT INVOLVEMENT 13
End of the Study 14
SELECTION AND WITHDRAWAL OF STUDY PARTICIPANTS 14
Recruitment 14
Eligibility criteria 14
Inclusion criteria 14
Exclusion criteria 15
Informed consent 15
STUDY TREATMENT AND REGIMEN 17
Sample size and justification 18
ADVERSE EVENTS 18
Definitions 18
Reporting of adverse events 19
TRANSPORT AND STORAGE OF THE TISSUES 20
LABORATORY ANALYSES 21
STATISTICS 21
Statistical analysis 21
Primary Outcome Measure 21
Secondary Outcome Measure 21
ETHICAL AND REGULATORY ASPECTS 22
Ethical Issues 22
Informed Consent and Participant Information 22
Withdrawal 22
RECORDS 23
Sample Labelling 23
Source documents 23
Direct access to source data / documents 23
Data Protection 23
QUALITY ASSURANCE & AUDIT 24
INSURANCE AND INDEMNITY 24
STUDY CONDUCT 24
STUDY DATA 24
RECORD RETENTION AND ARCHIVING 24
DISCONTINUATION OF THE STUDY BY THE SPONSOR 25
STATEMENT OF CONFIDENTIALITY 25

This protocol is confidential and the property of the University of Nottingham. No part of it may be transmitted, reproduced, published, or used by other persons without prior written authorisation from the University of Nottingham

PUBLICATION POLICY 25
STUDY FINANCES 25
Funding source 25
Participant stipends and payments 25
REFERENCES 25

ABBREVIATIONS

AE	Adverse Event
AR	Adverse Reaction
ALT	Alanine transaminase
ALP	Alkaline phosphatase
CI	Chief investigator
CPI	Checkpoint inhibitor
ChILI	Checkpoint inhibitors induced liver injury
CMV	Cytomegalovirus
CRF	Case Report Form
DILI	Drug-Induced Liver injury
EBV	Epstein-Barr virus
GCP	Good Clinical Practise
HIV	Human Immunodeficiency virus
iDILI	Idiosyncratic drug-induced liver injury
INR	International normalized ratio
irAE	Immune-related adverse events
ISF	Investigator Site File
NDD BRU	Nottingham Digestive Diseases, Biomedical Research Unit
NIHR	National Institute of Health Research
NUH	Nottingham University Hospitals
PI	Principal Investigator
PPI	Public Patient Involvement
R	Ratio
REC	Research Ethics committee
SAE	Serious Adverse Event
SAFE-T	Safer and faster Evidence based translation Consortium
SAR	Serious Adverse Reaction
SOP	Standard Operating Procedure
SUSAR	Suspected Unexpected Serious Adverse Reaction
TBL	Total bilirubin
TMF	Trial Master File
UAR	Unexpected Adverse Reaction
ULN	Upper limit of normal
WHO	World Health Organisation

This protocol is confidential and the property of the University of Nottingham. No part of it may be transmitted, reproduced, published, or used by other persons without prior written authorisation from the University of Nottingham

STUDY BACKGROUND INFORMATION AND RATIONALE

BACKGROUND

Immune checkpoint inhibitors (CPIs) have evolved over the last decade and treatment with a single CPI or a combination of CPIs has extended survival of patients with a variety of epithelial and lymphoid malignancies. The three major targets of CPIs are cytotoxic T-lymphocyte-associated antigen 4 (CTLA-4), programmed cell death protein 1 (PD-1), and its ligand, programmed cell death ligand 1 (PD-L1). They work by blocking immune suppressive ligand-receptor interactions which enhances the anticancer cytotoxic effects of lymphocytes. However, because checkpoint molecules are also involved in immune tolerance, unfavourable reactions to self-antigens potentially occur [1]. CPIs are associated with immune-related adverse events (irAE) in multiple organ systems including liver. Immune-mediated hepatitis is frequently observed in cancer patients treated with CPIs, up to 5% in single agent or 18% in combination therapies [2]. Checkpoint inhibitor-induced liver injury (ChILI) usually develops many weeks or even months after the start of the therapy, thus an early diagnosis of checkpoint inhibitor-induced hepatitis would help guide cancer therapy and management of drug-induced liver injury (DILI).

RATIONALE FOR CURRENT STUDY:

Despite remarkable progress in the treatment of several advanced malignancies with CPIs, immune mediated adverse events including checkpoint inhibitor-induced liver injury have been a real clinical challenge not only for physicians but also for cancer patients. This is due to the unpredictable nature of liver injury, lack of robust evidence to guide management and the potential consequences of stopping checkpoint inhibitor treatment. Current guidelines (European Society of Medical Oncology) suggest grading of the liver injury based on liver biochemistry test results: grade 1: alanine transaminase (ALT) or aspartate aminotransferase (AST) > 1 to 3 times upper limit of normal (ULN), grade 2 = ALT or AST >3 to 5 ULN, grade 3 = ALT or AST > 5 to 20 ULN and grade 4 = ALT or AST > 20 ULN [3]

Treatment guidelines for checkpoint inhibitor-induced liver injury are based on colitis and auto-immune hepatitis models with high dose corticosteroids being recommended for grade 3-4 liver injury or persistent grade 2 liver injury [3, 4]. However, steroids in cancer patients can cause serious adverse events and recent reports suggest that around 35% of grade 3/4 hepatitis regresses spontaneously without intervention [5, 6].

The immune-mediated liver injury induced by checkpoint inhibitors is much more frequent, a new and incompletely understood category of hepatotoxicity and distinct from other types of drug induced liver injury (DILI) such as direct or idiosyncratic DILI. Recent evidence suggests that ChILI is primarily mediated by CD8+ T cells, and it is associated with increased T cell activation and infiltration in the liver and with a characteristic histopathologic feature of necrotic/degenerative hepatocytes surrounded by ring-like T cell clusters [7]. This is different from direct chemically-induced, hepatocyte-intrinsic cell damage and death where hepatocyte cell death is typically the primary initiator of the inflammatory response [8]. This histologic feature is also different from autoimmune hepatitis or idiosyncratic DILI in humans [9].

This protocol is confidential and the property of the University of Nottingham. No part of it may be transmitted, reproduced, published, or used by other persons without prior written authorisation from the University of Nottingham

Candidate biomarkers are being investigated to identify diagnostic, prognostic and mechanistic biomarkers of idiosyncratic DILI [10, 11]. A series of robust genome wide association studies (GWAS) in the past decade have demonstrated the association of HLA alleles and other immune related gene polymorphism such as protein tyrosine phosphatase nonreceptor type 22 gene (*PTPN22*) with DILI [12-14].

A variety of approaches have been explored to identify biomarkers that are associated with efficacy in checkpoint inhibitor treated patients. These include immunophenotyping [15-17], cytoscore (cytokine profile) [18, 19], immune gene signature [20-26], TCR clonality analysis [27, 28], immunoscore, multiplex IHC of tissues and PD-L1 expression in tumour cells and associated immune cells [29-31]. These pharmacological changes can occur in peripheral circulation and/or tissues. While there have been some successes in CPI efficacy biomarkers, there are limited reports on identifying biomarkers for checkpoint inhibitor related adverse events. It is believed that the anti-tumor efficacy of CPI is mediated through its pharmacology- the enhanced T cell immune response against tumor antigens. Similarly, irAEs secondary to checkpoint inhibitors are also mediated via CPI pharmacology, likely through augmented immune response against self-antigens. And it appears there is an association between irAEs with efficacy in CPI treated patients [32]. Therefore, it is highly possible that biomarkers for CPI irAEs can also be identified in some if not all the approaches/assays described above. In fact, there has been some promise in identifying gene signatures for CPI related colitis [33]. In a monkey CPI-mediated irAE model, there was markedly increased proliferation and activation of CD4 and CD8 T cells and increased percentages of memory T cells, increased infiltration into tissues of predominant T cells, increased serum cytokines, and identified immune gene signatures such as T cell activation, APC activation and antigen presentation, and immune cell trafficking [34].

The data regarding the incidence and risk factors for this critical phenomenon is lacking. Therefore, we need 'in-depth phenotyping' together with data from control group exposed to CPIs to develop refined algorithms incorporating CPI-related factors, host genetic and environmental risk factors that would enable us to pre-empt ChILI. Deeply phenotyped cohorts with biological samples are essential for the development and validation of novel diagnostic/ prognostic markers [35] so that appropriate treatment guidelines can be developed for patient benefit.

Research has found that certain immune cell types are associated with drug induced liver injury (DILI) [36] and it was demonstrated that blood immune cell characteristics can indicate an autoimmune response to liver damage [37]. As CPIs modulate immune response, the immunological effect might play an important role in ChILI and predict its occurrence. Additionally, immunological analysis will enable researchers to establish markers which can be used to predict future DILI responses [38].

STUDY OBJECTIVES AND PURPOSE

PURPOSE

The purpose of the study is to identify the incidence of checkpoint inhibitor-induced liver injury (ChILI), the specific phenotypic features, and the risk factors associated with ChILI in addition to its relationship with other adverse reactions (especially immune-mediated colitis). It will enable us to obtain a better understanding of the mechanisms underlying ChILI and to develop methods of preventing ChILI and its consequences.

<u>Hypothesis:</u>

This protocol is confidential and the property of the University of Nottingham. No part of it may be transmitted, reproduced, published, or used by other persons without prior written authorisation from the University of Nottingham

We hypothesise that checkpoint inhibitor-induced liver injury (ChILI) is determined by factors that modulate cellular and immunological response to the development of ChILI and can be recognised by a combination of markers.

PRIMARY OBJECTIVE

To collect, store and analyse biological samples and clinical data form two prospective longitudinal cohorts, one includes cancer patients undergoing checkpoint inhibitor therapy and another cohort of patients who developed checkpoint inhibitor-induced liver injury (ChILI).

Proposal Aims:

1. To establish the incidence of ChILI and other immune-related adverse events over a period of 6-14 weeks following checkpoint inhibitor therapy.
2. To identify and characterise biomarkers associated with the diagnosis of ChILI by comparing biomarkers of patients who developed ChILI with patients who had CPI treatment without developing liver injury with follow up to 14 weeks.

SECONDARY OBJECTIVES

1. To study the natural history of patients treated with checkpoint inhibitors after developing ChILI.
2. To identify specific phenotypic features associated with ChILI compared to idiopathic auto-immune hepatitis and DILI due to other drugs. This includes histological characteristics, genetic, biochemical and immunological characteristics.
3. To determine association between biomarkers, clinical features and development of ChILI.

STUDY DESIGN

STUDY CONFIGURATION

It is a multi-centre study which contains four parts, two parallel longitudinal prospective cohorts as outlined below (cohort of patients before and after CPI treatment and cohort of patients who already had treatment and developed liver injury), a cross sectional case control study to compare biomarkers and a liver histology study.

Part 1. CPI cohort study (before & after CPI therapy)
We will prospectively develop a cohort of oncology patients **(cohort study)**, with malignant melanoma, renal cell carcinoma, non-small cell lung cancer or any other cancer, receiving single or combination therapy using checkpoint inhibitors (CPIs). Two visits will be undertaken (prior starting CPI therapy and 6-14 weeks after treatment, when collection and storage of biological samples and data (medical, demographic and clinical) will be obtained. Timing of ChILI ranges from 1 – 49 weeks following treatment with a peak around 6 – 14 weeks [5] ,

Part 2. ChILI cohort
We will recruit patients who are already on CPI therapy and have developed liver injury (ChILI) which fits DILI criteria (*ALT > 5 ULN OR ALT > 3 ULN plus bilirubin > 2 ULN OR alkaline phosphatase (ALP) > 2 ULN* with accompanying elevations of gamma-

This protocol is confidential and the property of the University of Nottingham. No part of it may be transmitted, reproduced, published, or used by other persons without prior written authorisation from the University of Nottingham

glutamyl transferase (GGT) in the absence of known bone metastases driving the rise in ALP level). We will follow up the natural history of ChILI in these patients for 1 month after enrolment. Biological samples will be taken from these patients at 3 time points (date of reaction, 1 week after and 1 month after).

<u>Part 3. Cross-sectional case/control study (comparing patients in parts 1 and 2)</u>
We will run a cross-sectional case control study comparing biological samples of patients in the cohort study who did not develop ChILI after 6-14 weeks of CPI treatment (controls) with patients in the second study who developed ChILI (cases). A small percentage of patients in the cohort study (3-18%) will develop ChILI and will be added to the cases; they will have 4 visits in total (one prior CPI treatment and 3 visits following reaction). We will compare samples from visit 2 in controls (after 6-14 weeks of treatment without developing ChILI) and visit 1 in cases (time of reaction).

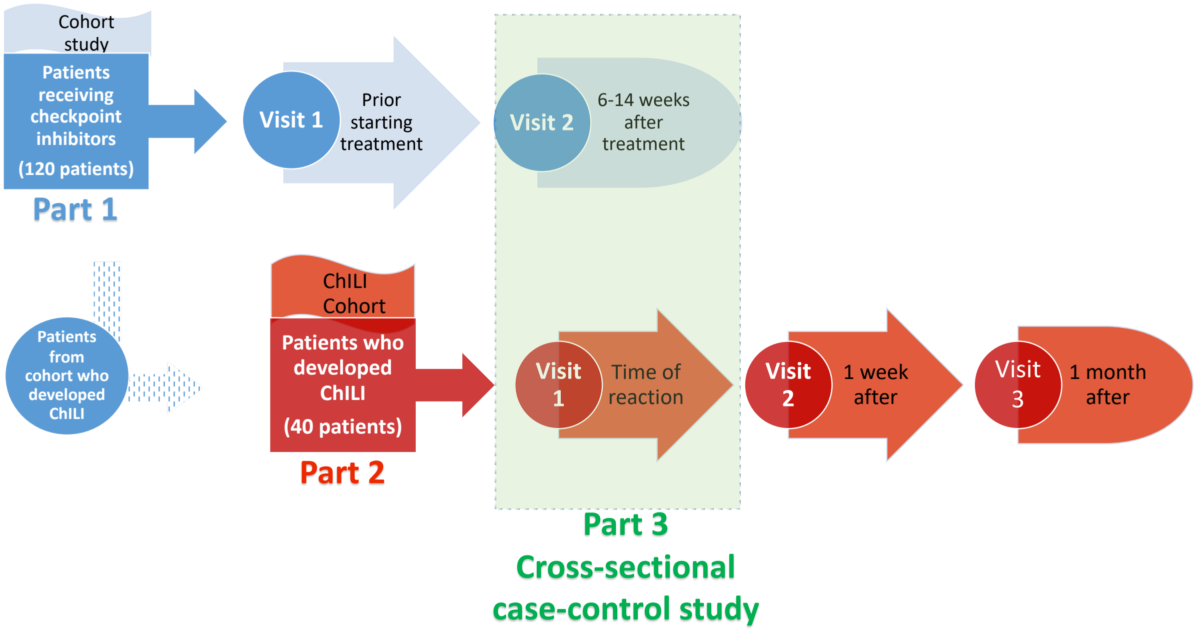

<u>Part 4. Liver Histology Study</u>
We aim to compare histological characteristics of ChILI with idiopathic auto-immune hepatitis (AIH) and DILI due to other drugs. We expect to obtain 10 liver biopsies from patients with ChILI and perform histological comparison with up to 25 liver biopsies of DILI and 50 biopsies of auto-immune hepatitis available retrospectively and prospectively through other REC/HRA-approved research studies (where consent is given for researchers to access surplus diagnostic material) and requested from research tissue banks (such as NDDC BRU Research tissue bank REC Ref 19/WA/0288 IRAS 269958 and Transbioline Prospective European drug-induced liver injury registry (PRO-EURO DILI) REC 15/YH/0294 (based within the University of Nottingham).
In order to obtain sufficient samples which meet histopathologist-confirmed disease definitions, we will also request surplus FFPE tissue, remaining in archives after clinical diagnosis, from the pathology department at Nottingham University Hospitals NHS Trust. (NUHT) Samples relating to cases coded as confirmed DILI or AIH in medical notes will be, randomly selected and released in fully anonymised

This protocol is confidential and the property of the University of Nottingham. No part of it may be transmitted, reproduced, published, or used by other persons without prior written authorisation from the University of Nottingham

format (with no associated personal data) by the clinical pathology translational research team to the study research team We will also approach other research tissue banks (e.g. Birmingham or Liverpool) to request further tissue samples required if necessary to complete the study since samples available may be limited. Samples will be FFPE-preserved liver tissue for histology analysis by pathologist and material for phenotypic analysis (e.g. histochemical or genetic/expression). Fresh-frozen liver tissue samples will also be analysed where available. This will enable us to develop a clear picture of cellular changes in the liver associated with the natural history of ChILI.

Study Centres:

- NIHR Biomedical Research Centre, Nottingham University Hospitals NHS Trust and University of Nottingham, Queens medical Centre, NG7 2UH

NHS secondary care hospital sites in the UK (Including Scotland)

Primary endpoint

Following acquisition of clinical samples and collation of associated comprehensive demographic, phenotypic and clinical data collected from a total of:
40 patients who develop checkpoint inhibitors-induced liver injury (ChILI) and
120 patients with cancer are treated with checkpoint inhibitors, we aim to:

1. Measure the incidence of liver injury secondary to checkpoint inhibitors and other adverse events.
2. Identify and characterise biomarkers associated with ChILI.

Secondary endpoint

1. Deep phenotyping of patients who developed ChILI, studying their natural history and comparing their characteristics with idiopathic auto-immune hepatitis and DILI due to other drugs. This includes histological characteristics, genetic, biochemical and immunological characteristics.
2. Studying the association between biomarkers, clinical features and development of ChILI.

STUDY MANAGEMENT

Participants enrolled prospectively will be asked to give consent for their samples to be collected, stored and used for the purposes of this study. Clinical and phenotypic data will be collected.

Blood collected will be used for genetic analyses of genetic variants (not genetic testing for clinical genetic disease diagnostics) and for immune cell characterisation. The total volume of blood taken for these samples will not exceed 80ml.
We propose to prepare peripheral blood mononuclear cells (PBMCs) for phenotypic analysis such as scRNAseq and immunological cell-type analyses. Plasma and serum will be prepared from blood samples after collection and analysed or stored at -80°C for subsequent analysis of biomarkers, metabolites and other cellular components. We plan assessment of proposed circulating biomarkers including cytokines, microRNAs (miR-122, miR-4270 and miR-4463)[39], total cytokeratin 18 (K18), macrophage colony-stimulating factor receptor (MCSFR) [11], and any others identified in subsequent publications. Some of these molecules may be quantified by specialist laboratories via service agreements. Urine

This protocol is confidential and the property of the University of Nottingham. No part of it may be transmitted, reproduced, published, or used by other persons without prior written authorisation from the University of Nottingham

biomarkers and metabolites will be evaluated and metabolites and microbiome assessed from stool samples.

We will also collect liver biopsy material surplus from clinical care where available only if taken as part of usual patient treatments for their condition. We plan to use **available** liver biopsies, up to 25 of DILI and 50 of AIH, from other HRA-approved research studies, tissue banks and the histopathology archive at NUHT to examine the cellular features of disease and provide phenotypic details of changes within liver tissues that accompany the disease development.

We will also request consent for any residual samples after the study is complete to be transferred to a research tissue bank or stored under the University HTA license and used for future research by other researchers.

In this case, samples will be stored at the NDDC BRU Research Tissue bank (REC. Ref: 19/WA/0288; IRAS 269956) and made available through the access committee.
The Chief Investigator has overall responsibility for the study and shall oversee all study management.

The data custodian will be the Chief Investigator. Anonymised data will be shared with researchers at Pfizer where appropriate for analysis of research findings.

DURATION OF THE STUDY AND PARTICIPANT INVOLVEMENT

STUDY DURATION:

Five years.

PARTICIPANT DURATION:

- Checkpoint inhibitors cohort group (part one on diagram): <u>two visits:</u>

    1. Prior starting checkpoint inhibitors

    2. After 6-14 weeks of treatment if no ChILI develops.

- ChILI group (part two on diagram): <u>three visits:</u>

    1. At time of reaction, defined as: *ALT > 5 ULN* <u>*OR*</u> *ALT > 3 ULN plus bilirubin > 2 ULN OR alkaline phosphatase (ALP) > 2 ULN* with accompanying elevations of gamma-glutamyl transferase in the absence of known bone metastases driving the rise in ALP level.
    2. One week later (2-14 days after Visit 1).

    3. One month later (30 ± 7 days after the Visit 1).

In cases where ChILI develops in the checkpoint inhibitors cohort group (part one), there will be a total of 4 visit – prior starting CPI, time of reaction, 1 week after reaction and 1 month after reaction

This protocol is confidential and the property of the University of Nottingham. No part of it may be transmitted, reproduced, published, or used by other persons without prior written authorisation from the University of Nottingham

End of the Study

The end of the study will be the last visit of the last participant.

Laboratory analyses will continue for 12 months.

SELECTION AND WITHDRAWAL OF STUDY PARTICIPANTS

Recruitment

The study will be done in the secondary care setting, across NHS sites in the UK (Including Scotland)where patients with cancer on CPIs are followed up and likely to present following a liver injury. Participants will be mainly recruited from oncology clinics and wards or from emergency departments when they present with acute symptoms. The initial approach will be from a member of the patient's usual care team (which may include the investigator or research team), and information about the study will be on display in the relevant clinical areas. Further visits will take place at the ward if patients remain hospitalised, at clinic following oncology follow up or at the Research Centres wherever is more convenient to the patients.

The investigator or their nominee, e.g. from the research team or a member of the participant's usual care team, will inform the participant or their nominated representative (other individual or other body with appropriate jurisdiction), of all aspects pertaining to participation in the study.

If needed, the usual hospital interpreter and translator services will be available to assist with discussion of the trial, the participant information sheets, and consent forms. The consent forms and information sheets will not be available printed in other languages.

It will be explained to the potential participant that that entry into the trial is entirely voluntary and that their treatment and care will not be affected by their decision. It will also be explained that they can withdraw at any time, but attempts will be made to avoid this occurrence. In the event of their withdrawal it will be explained that their data collected so far cannot be erased and we will seek consent to use the data in the final analyses where appropriate. Remaining tissue samples can be destroyed if the participant so wishes. We will explain to the participant that if they decide to withdraw after a long period of time, the samples may already have been used and we cannot recall samples or information from researchers if this is the case.
The Principal Investigator may also remove a subject if, in his / her opinion, it is in the best interests of the subject. If a patient permanently withdraws from the study, or is lost to follow-up, the reason will be recorded.

Eligibility criteria

Inclusion criteria

Both patient groups and control group:
Aged 16 years and over
Able to give written informed consent OR

This protocol is confidential and the property of the University of Nottingham. No part of it may be transmitted, reproduced, published, or used by other persons without prior written authorisation from the University of Nottingham

Potential participants who have developed encephalopathy related to ChILI as a response to checkpoint inhibitor therapy, who lack capacity to give written informed consent and have a consultee (personal or nominated) – for ChILI patient group only

ChILI Patients:
Adults with cancer receiving checkpoint inhibitors (CTLA-4, PD-1 or PD L1 inhibitor) as monotherapy or combination (without chemotherapy) and developed acute liver injury secondary to checkpoint inhibitor and meeting the following criteria will be included:

1. Meets one of the following analytical thresholds at enrolment (visit 1)
    - Alanine transaminase (ALT) exceeding 5 times upper limit of normal (ULN) OR
    - ALT exceeding 3 times ULN plus bilirubin exceeding 2 times ULN OR
    - Alkaline phosphatase (ALP) exceeding 2 times ULN with accompanying elevations of gamma-glutamyl transferase in the absence of known bone metastases driving the rise in ALP level
2. Absence of other known causes of liver injury after detailed investigations

Patients who developed ChILI but did not meet the above criteria at enrolment or who were found to have different cause for their liver injury after further investigations will be excluded from analysis

Control Group:
Consecutive patients with cancer who have clinical indication to start checkpoint inhibitors will be approached for consent to be enrolled in the study. We will enrol 120 consecutive patients who will be identified as controls. A small proportion of patients will develop ChILI following their checkpoint inhibitor treatment and will be classified as cases.

Exclusion criteria

Anyone with any of the following conditions will not be included in the study:

- Patients who are treated with cytotoxic chemotherapy concurrently with checkpoint inhibitors.
- On the judgement of CI that the person has certain alternative explanation to the acute event (rather than ChILI).

Informed consent

We will obtain informed consent from participants or their consultee in case they lack capacity to consent. The Informed Consent Form will be signed and dated before they enter the trial. The Investigator will explain the details of the trial and provide a Participant Information Sheet/Consultee Information Sheet, ensuring that the participant/consultee has sufficient time to consider participating or not and ask questions about the study.

The process for obtaining participant informed consent will be in accordance with the REC guidance, and Good Clinical Practice (GCP) and any other regulatory requirements that might be introduced. Since patients who develop acute checkpoint inhibitors liver injury can often present in emergency departments with sudden onset of severe symptoms, a long period (e.g. 24h) to consider consent is often difficult to provide in order to recruit patients while symptomatic and without asking patients to make additional hospital visits. Therefore, where appropriate patients will be given a reasonable time period (at least 1 hour without disturbance), to read the patient information sheet and consider enrolment before being approached for consent by the research team. Appropriate steps will be taken

This protocol is confidential and the property of the University of Nottingham. No part of it may be transmitted, reproduced, published, or used by other persons without prior written authorisation from the University of Nottingham

to ensure that the patient is able to discuss participation in the study with those not involved in the research. The investigator or their nominee and the participant or other legally authorised representative shall both sign and date the Consent Form before the person can participate in the study.

The participant will receive a copy of the signed and dated forms and the original will be retained in the study records. A second copy will be filed in the participant's medical notes and a signed and dated entry made in the notes that informed consent was obtained for the study. Due to COVID-19 pandemic, we will also consider electronic consent (e-consent) through the secure website (REDCap) as a method of obtaining informed consent to reduce face to face contacts with patients.

The decision regarding participation in the study is entirely voluntary. The investigator or their nominee shall emphasize to them that consent regarding study participation may be withdrawn at any time without penalty or affecting the quality or quantity of their future medical care, or loss of benefits to which the participant is otherwise entitled. No study-specific interventions will be done before informed consent has been obtained.

The investigator will inform the participant of any relevant information that becomes available during the course of the study, and will discuss with them, whether they wish to continue with the study. If applicable they will be asked to sign revised consent forms.

If the Consent Form is amended during the study, the investigator shall follow all applicable regulatory requirements pertaining to approval of the amended Consent Form by the REC and use of the amended form (including for on-going participants).

Participants who lack capacity to consent to research

Due to the nature of the study some potential participants may lack capacity to give informed consent. This is especially likely in the case of hepatic encephalopathy, which is the occurrence of confusion, altered level of consciousness, and coma as a result of liver failure. It is important that we try to enrol such patients where possible into this study, as research into this area may be of benefit to future patients with this condition. As the study has minimal risk to the patient, we believe that the benefits of enrolling such patients outweigh the potential risk.

We have referred to the mental capacity act in our decision to enrol patients who lack capacity. There are a number of provisions specific to research that we have taken into account. It is noted that the research in question will be connected with the impairing condition affecting the participant. We will only seek consent from those who lack capacity in a way that is related to their liver injury. We therefore will not seek consent from those who would have been likely to lack capacity before any liver injury has occurred. We will therefore not be enrolling patients who are believed to have a "double impairment". Secondly, we believe that research of equal effectiveness cannot be carried out if confined to participants with capacity, as, the most severe cases of the condition we are researching, can cause impairments. We believe that it is important to include the most severe cases in our research. Thirdly, although our research does not directly benefit the patient, this research will provide knowledge of the causes or treatment of others with the same condition, and involves negligible risk to the participant, does not interfere significantly with freedom of action or privacy, and is not unduly invasive or restrictive.

If the PI or Investigator has reason to suspect that a person may lack capacity, that individual will be assessed according to normal local hospital procedures. If the person is found to lack capacity then the research team will ask someone close to that person if they would consider acting as their personal consultee, e.g. next of kin, a relative, spouse, close friend. The personal consultee should be someone the person who lacks capacity would trust with important decisions about their welfare.

This protocol is confidential and the property of the University of Nottingham. No part of it may be transmitted, reproduced, published, or used by other persons without prior written authorisation from the University of Nottingham

If the PI or Investigator and research team are unable to identify/appoint a suitable personal consultee then efforts will be made to nominate a health professional who is unconnected with the research project to act as a professional consultee. We will firstly ask a member of the standard medical care team of the person who lacks capacity if they would consider taking on the role. The professional consultee will be required to perform the same role as a personal consultee in advising the research team but they may not know the person who lacks capacity and therefore may be required when attempting to determine what the person's wishes and feelings about the research would be if they had capacity, seek the views of any family, friends/carers of the person who lacks consent, or seek other professional colleagues with an interest in the person's welfare or condition. We will continue to assess capacity throughout the research and will seek consent as soon as feasible.

All consultees (personal and professional) will receive a copy of the consultee information sheet to read. If and when the participant regains capacity, consent will be sought from the participant retrospectively, and will be withdrawn from the study if consent is not given.

We currently have no sites or plans to recruit from sites in Northern Ireland. If this was to occur, we are aware that Northern Ireland follow different legislation and a different process would need to be followed regarding the documentation. We would ensure that this process was followed correctly. We are currently seeking approval to recruit these patients in England and Wales only. We plan to put a future application to a Scotland A REC in order to allow us to recruit patients who lack capacity in Scotland.

STUDY TREATMENT AND REGIMEN

Suspected ChILI patients:

Biological samples will be collected from each enrolled patient on three occasions. These visits will take place as soon as possible following acute presentation; there will be a time lag of up to 2 weeks between identification of an episode of ChILI by the team responsible for the clinical care of the patients and the patient giving consent to take part in the study. Patients' blood tests must meet the eligibility criteria on the day they are enrolled (Day 0, visit 1) in order to participate in the ChILI study. Visit 2 will be 2-14 days from Visit 1. Visit 3 will be 30 ± 7 days after the Visit 1.

As far as possible, research bloods will be collected at the same time as bloods for clinical care of the patient and visits arranged to coincide with hospital appointments. Preferred visit times are given below but visits outside the times will not be considered as a protocol deviation.

Appendix 1 lists the standard clinical care analyses (including ultrasound, viral hepatitis screen, autoantibody screen, haematology and blood chemistry), and research assessments which will be carried out at each visit.

For those who are in-patients (hospitalised at the time of acute ChILI) samples will be collected as part of clinical care and additional samples collected for research purposes.

At each visit a delegated member of the research team will collect:

- Up to 80ml of blood. See most recent study presentation and/or sample collection SOP for details.
- Up to **20 ml of urine if possible**
- A Stool sample will also be collected (if possible).

When considered appropriate by the clinical team, an additional optional 30ml blood will be requested at one or two visits. This will be collected in EDTA anti-coagulated tubes and

This protocol is confidential and the property of the University of Nottingham. No part of it may be transmitted, reproduced, published, or used by other persons without prior written authorisation from the University of Nottingham

transferred directly to the research lab for processing immediately where this facility is available.

Patients may be consented and samples collected at their home if appropriate and with permission of the PI. Nursing staff would be required to follow their Trust's lone working policy and code of practice.

All biological samples will be processed according to the standard operating procedures labelled and stored at -80°C in Nottingham Digestive Diseases Centre. If a liver biopsy is obtained for clinical reasons, any samples available (when the liver biopsy core is >25 mm) will be collected and stored for research purposes. Fresh liver tissue will be snap frozen in liquid nitrogen or placed in RNA later prior to storage at -80°C. (We will also aim to obtain and store any archived liver sections that were taken as part of clinical care). The liver biopsy specimens will be reviewed centrally by Dr Philip Kaye.

Control group

Prior staring checkpoint inhibitor treatment, patients will have a baseline visit when biological samples will be collected (blood, stool and urine sample) and processed as described for ChILI patients visit 1. Further visit will take place 6-14 weeks after starting CPI treatment. Demographic data and medical and pharmacological history will also be collected as outlined in the CRF

Sample size and justification

This is a mechanistic study to document the disease natural history rather than evaluate performance of specific biomarkers. As this uses specialist methods, we will only be able to recruit from a small number of suitable sites near to analytical facilities so sample size is limited by availability of suitable participants. Due to rarity of checkpoint inhibitors induced liver injury, incidence rate are between 3-18 % of patients receiving CPI, we aim to enrol 40 cases and 120 controls who received CPI without developing liver injury over 3 years. This is feasible at this centre based on current records of CPI treatment.

ADVERSE EVENTS

Definitions

An adverse event is any unfavourable and unintended sign, symptom, syndrome or illness that develops or worsens during the period of observation in the study.

An AE does include a / an:

1. Exacerbation of a pre-existing illness.

2. Increase in frequency or intensity of a pre-existing episodic event or condition.

3. Continuous persistent disease or symptoms present at baseline that worsen following the start of the study.

A Serious Adverse Event (SAE) is any untoward and unexpected medical occurrence or effect that:

- Results in death
- Is life-threatening – refers to an event in which the subject was at risk of death at the time of the event; it does not refer to an event which hypothetically might have caused death if it were more severe
- Requires hospitalisation, or prolongation of existing inpatients' hospitalisation

This protocol is confidential and the property of the University of Nottingham. No part of it may be transmitted, reproduced, published, or used by other persons without prior written authorisation from the University of Nottingham

- Results in persistent or significant disability or incapacity
- Is a congenital anomaly or birth defect

Important medical events that may not result in death, be life-threatening, or require hospitalisation may be considered a serious adverse event when, based upon appropriate medical judgment, they may jeopardize the patient or participant and may require medical or surgical intervention to prevent one of the outcomes listed in this definition

AE doesn't include a/ an:

1. Exacerbation of a pre-existing illness or progression of underlying malignancy.
2. Increase in frequency or intensity of a pre-existing episodic event or condition.
3. Condition detected or diagnosed after medicinal product administration even though it may have been present prior to the start of the study.
4. Continuous persistent disease or symptoms present at baseline that worsen following the start of the study.
5. Medical or surgical procedure (e.g., surgery, endoscopy, tooth extraction, transfusion); but the condition that lead to the procedure is an AE.
6. Pre-existing disease or conditions present or detected at the start of the study that did not worsen.
7. Situations where an untoward medical occurrence has not occurred (e.g., hospitalisations for cosmetic elective surgery, social and / or convenience admissions).
8. Disease or disorder being studied or sign or symptom associated with the disease or disorder unless more severe than expected for the participant's condition.
9. Overdose of concurrent medication without any signs or symptoms.

All adverse events will be assessed for seriousness and expectedness:

A distinction is drawn between serious and severe AEs. Severity is a measure of intensity whereas seriousness is defined using the criteria above. Hence, a severe AE need not necessarily be serious.

Reporting of adverse events

All adverse events (as outlined below) should be reported to the PI and CI. Depending on the nature of the event the reporting procedures below should be followed. Any questions concerning adverse event reporting should be directed to the Chief Investigator in the first instance.

Non-serious Adverse Event (NSAE)

All such events, whether expected or not, should be recorded.

This is a case control study and doesn't include any clinical or experimental interventions other than blood sampling. In addition, natural history of DILI includes progressive worsening of the liver injury even after offending agent/ medication/ drug has been withdrawn. Acute liver failure and death are recognised complication. Therefore, while we will record accurately the severity of DILI formally based on the international consensus criteria [40].

Serious AEs

This protocol is confidential and the property of the University of Nottingham. No part of it may be transmitted, reproduced, published, or used by other persons without prior written authorisation from the University of Nottingham

An SAE form should be completed and faxed to the Chief Investigator within 24 hours.
A Serious Adverse Event (SAE) is any adverse event occurring following study mandated procedures, having received the treatment or intervention that results in any of the following outcomes. However, acute liver failure, liver transplantation and death are recognised consequences of DILI and these will be recorded as part of the outcome of DILI as recommended by consensus criteria [40]. Therefore, following will NOT be considered as SAE:

1. Liver related death
2. Acute liver failure
3. Liver transplantation
4. Inpatient hospitalisation or prolongation of existing hospitalisation
5. A disability / incapacity

In the current study we will be performing venepunctures for blood sampling. So, we will report major bleeding leading to hypotension, drop in Hb, requirement of resuscitation, transfusion or hospital admission as adverse events. All SAEs should be reported to the North West - Haydock Research Ethics Committee where in the opinion of the Chief Investigator, the event was:
- 'related', ie resulted from the administration of any of the research procedures; and
- 'unexpected', ie an event that is not listed in the protocol as an expected occurrence

Reports of related and unexpected SAEs should be submitted within 15 days of the Chief Investigator becoming aware of the event, using the COREC SAE form for non-IMP studies.

Local investigators should report any SAEs as required by their Local Research Ethics Committee and/or Research & Development Office.

TRANSPORT AND STORAGE OF THE TISSUES

Samples collected prospectively will be stored in a linked anonymised format and labelled using a combination of study reference, unique study identifier and cross referenced with location code numbers to permit accurate linkage to study data and the consent form. Liver tissue from patients with AIH and DILI collected from the archive of the histology department at Nottingham University Hospitals NHS Trust will be fully anonymised FFPE preserved samples from the archive will be returned or disposed.

Samples for NHS pathology analysis will be labelled in accordance with local NHS procedures.

Serum, plasma, whole blood, tissue, purified cells (PBMCs), urine, and stool samples will be stored in aliquots at -80°C in the Nottingham Digestive Diseases Centre within University of Nottingham, in the Queens Medical Centre Campus, held under HTA licenced premises (Designated Individual: James Dixon, Licence Number 12265). When the study closes, samples will be transferred to NDDC BRU research tissue bank. Samples will be shipped following HTA and International laws with appropriate agreements in place. Samples including liver tissue may be sent to our partners, Pfizer in USA and specialist analytical centres worldwide (contract in place).

This protocol is confidential and the property of the University of Nottingham. No part of it may be transmitted, reproduced, published, or used by other persons without prior written authorisation from the University of Nottingham

The master database will be held by the CI and designated individuals in a password encrypted file.

Where participants do not agree to the future use of the samples they will be destroyed in accordance with the Human Tissue Act, 2004.

LABORATORY ANALYSES

Each participating NHS hospital site will carry out all routine laboratory analysis as part of normal patient care. Blood will be processed in Nottingham Digestive Diseases Centre or Nottingham BRC lab for storage of serum and plasma as outlined in the SOP. Data and all appropriate documentation will be stored for a minimum of 25 years after the completion of the study, including the follow-up period.
We will collate detailed and comprehensive clinical and demographic data using electronic case report form (CRF) on the University of Nottingham REDCap platform. Data from analysis of biological samples (i.e. biomarkers) and histology will be collated and linked to clinical characteristics on study databases to achieve in-depth phenotyping.

STATISTICS

Statistical analysis

- We will calculate the incidence of ChILI and other irAE in the cohort group.
- We will describe demographic and clinical data for the enrolled participants using descriptive statistics, mean ± standard deviation (SD) for continuous measurements and frequencies and percentiles for categorical data. We will compare patients' pathological and clinical characteristics using Chi-square test for categorical variables and Wilcoxon/Kruskal-Wallis test for the continuous variables. $P < 0.05$ will be considered statistically significant.
- We will assess the accuracy of the biomarkers for ChILI detection and predicting prognosis using receiver operating characteristic (ROC) curve and the area under the ROC curve (AUC) with 95% confidence interval (CI).
- We will assess the correlation of biomarkers with ALT using Pearson's r coefficient.

Primary Outcome Measure

1. To estimate the incidence of checkpoint inhibitor-induced liver injury (ChILI) and other immune-mediated adverse reactions.
2. To identify and characterise biomarkers associated with the diagnosis of ChILI

Secondary Outcome Measure

1. To identify risk factors and phenotypic features associated with ChILI and its association with other immune mediated adverse reactions.

This protocol is confidential and the property of the University of Nottingham. No part of it may be transmitted, reproduced, published, or used by other persons without prior written authorisation from the University of Nottingham

2. To study the association between biomarkers, clinical features and development of ChILI.
3. To compare histological characteristics of ChILI with idiopathic auto-immune hepatitis and DILI due to other drugs.

ETHICAL AND REGULATORY ASPECTS

Ethical Issues

The study will not be initiated before the protocol, informed consent forms and participant information sheets have received approval / favourable opinion from a Research Ethics Committee (REC). Should a protocol amendment be made that requires REC approval, the changes in the protocol will not be instituted until the amendment and revised informed consent forms and participant information sheets have been reviewed and received approval / favourable opinion from the REC. A protocol amendment intended to eliminate an apparent immediate hazard to participants or researchers may be implemented immediately providing that the REC is notified as soon as possible and an approval is requested. Minor protocol amendments only for logistical or administrative changes may be implemented immediately; and the REC will be informed.

The study will be conducted in accordance with the ethical principles that have their origin in the Declaration of Helsinki, 1996; the principles of Good Clinical Practice and Good Laboratory Practice, the UK Department of Health Policy Framework for Health and Social Care, 2017 and in accordance with the Human Tissue Act, 2004.

Informed Consent and Participant Information

The process for obtaining participant informed consent or consultee informed consent will be in accordance with the REC guidance, and Good Clinical Practice (GCP) and any other regulatory requirements that might be introduced. The investigator and the participant or their consultee shall both sign and date the Consent Form before the person can participate in the study.

The participant will receive a copy of the signed and dated forms and the original will be retained in the Study Master File. A second copy will be filed in the participant's medical notes and a signed and dated note made in the notes that informed consent was obtained for the study. Due to COVID-19 pandemic, we will also consider electronic consent (e-consent) through the secure website (REDCap) as a method of obtaining informed consent to reduce face to face contacts with patients.

The decision regarding participation in the study is entirely voluntary. The investigator or their nominee shall emphasize to them that consent regarding study participation may be withdrawn at any time without penalty or affecting the quality or quantity of their future medical care, or loss of benefits to which the participant is otherwise entitled. No study-specific interventions will be done before informed consent has been obtained.

If the Consent Form is amended during the study, the investigator shall follow all applicable regulatory requirements pertaining to approval of the amended Informed Consent Form by the REC and use of the amended form (including for ongoing participants).

Withdrawal

The investigator will explain that participation in the study is entirely voluntary and that the participant will retain the right to withdraw at any time without prejudice by simply informing

This protocol is confidential and the property of the University of Nottingham. No part of it may be transmitted, reproduced, published, or used by other persons without prior written authorisation from the University of Nottingham

the investigator. Should samples and data already have been obtained we will inform the participant that we would still like to use these samples and data and this will be indicated in the information sheet and consent form. Remaining tissue samples will be destroyed if the participant so wishes.

RECORDS

Each participant will be assigned a trial identity code number for use on the CRFs, samples, consent forms and other trial documents and the electronic database.

CRFs will be treated as confidential documents and held securely in accordance with regulations. The investigator will make a separate confidential record of the participant's name, date of birth, local hospital number or NHS number, and Participant Study Number (the Study Recruitment Log), to permit identification of all participants enrolled in the trial, in accordance with regulatory requirements and for follow-up as required

CRFs shall be restricted to those personnel approved by the Chief or local Principal Investigator and recorded on the 'Study Delegation Log.'

All paper forms shall be filled in using black ballpoint pen. Errors shall be lined out but not obliterated by using correction fluid and the correction inserted, initialled and dated.

The Chief or local Principal Investigator shall sign a declaration ensuring accuracy of data recorded in the CRF.

Sample Labelling

Each participant will be assigned a trial identity code number for use on the samples. Samples for NHS pathology analysis will be labelled in accordance with local NHS procedures.

Source documents

Source documents shall be filed at the investigator's site and may include but are not limited to, consent forms, current medical records, laboratory results and records. A CRF may also completely serve as its own source data. Only trial staff as listed on the Delegation Log shall have access to trial documentation other than the regulatory requirements listed below.

Direct access to source data / documents

Study documents, including progress notes and copies of laboratory and medical test results shall made be available at all times for review by the Chief Investigator, Sponsor's designee and inspection by relevant regulatory authorities.

Data Protection

All study staff and investigators will endeavour to protect the study participants' rights to privacy and informed consent, and will adhere to the Data Protection Act, 2018. Only the minimum required information for the purposes of the study shall be collected. Documents will be held securely, in a locked room, or locked cupboard or cabinet. Access to the information will be limited to the study staff and investigators and relevant regulatory authorities (see above). Computer held data including the study database will be held securely and password protected. All clinical data collected from participants will be stored on a secure dedicated web server on the University of Nottingham REDCap platform. Access will be restricted by user identifiers and passwords (encrypted using a one-way encryption method).

Information about the study in the participant's medical records / hospital notes will be treated confidentially in the same way as all other confidential medical information.

This protocol is confidential and the property of the University of Nottingham. No part of it may be transmitted, reproduced, published, or used by other persons without prior written authorisation from the University of Nottingham

Only anonymised data will be shared outside the institution. A separate collaboration agreement will be in place describing the data to be shared with Pfizer. All data transfer outside the institution will be anonymised and via password protected encrypted files.

QUALITY ASSURANCE & AUDIT

INSURANCE AND INDEMNITY

Insurance and indemnity for study participants and NHS staff is covered within the NHS Indemnity Arrangements for clinical negligence claims in the NHS, issued under cover of HSG (96)48. There are no special compensation arrangements, but study participants may have recourse through the NHS complaints procedures.

The University of Nottingham as research Sponsor indemnifies its staff, research participants and research protocols with both public liability insurance and clinical trials insurance. These polices include provision for indemnity in the event of a successful litigious claim for proven non-negligent harm.

STUDY CONDUCT

Study conduct may be subject to systems audit of the study files for inclusion of essential documents; permissions to conduct the study; CVs of study staff and training received; local document control procedures; consent procedures and recruitment logs; adherence to procedures defined in the protocol (e.g. inclusion / exclusion criteria, timeliness of visits); and equipment calibration logs.

STUDY DATA

Monitoring of study data shall include confirmation of informed consent; source data verification; data storage and data transfer procedures; local quality control checks and procedures, back-up and disaster recovery of any local databases and validation of data manipulation. The Chief Investigator, or where required, a nominated designee by the sponsor or funder, shall carry out monitoring of study data as an ongoing activity.

Data entries will be verified by inspection against the source data. A sample (10% or as per the study risk assessment) will be checked on a regular basis for verification of all entries made. In addition, the subsequent capture of the data on any study databases will be checked. Where corrections are required these will carry a full audit trail and justification.
Study data and evidence of monitoring and systems audits will be made available for inspection by the regulatory authority as required.

RECORD RETENTION AND ARCHIVING

In compliance with the DH Research Governance Framework guidelines, the Human Tissue Act and in accordance with the University of Nottingham Code of Research Conduct and Research Ethics, the Chief Investigator will maintain all records and documents of the study. These will be retained for at least 7 years or for longer if required. We will be seeking consent to collate, store and use samples and data for a minimum of 10 years. If the responsible investigator is no longer able to maintain the study records, a second person will be nominated to take over this responsibility.

This protocol is confidential and the property of the University of Nottingham. No part of it may be transmitted, reproduced, published, or used by other persons without prior written authorisation from the University of Nottingham

The study documents held by the Chief Investigator on behalf of the Sponsor shall be finally archived at secure archive facilities at the University of Nottingham. This archive shall include all study databases and associated meta-data encryption codes.

DISCONTINUATION OF THE STUDY BY THE SPONSOR

The Sponsor reserves the right to discontinue this study at any time for failure to meet expected enrolment goals, for safety or any other administrative reasons.

STATEMENT OF CONFIDENTIALITY

Individual participant medical information obtained as a result of this study is considered confidential and disclosure to third parties is prohibited with the exceptions noted above. Participant confidentiality will be further ensured by utilising identification code numbers to correspond to study data in the computer files.

Data generated as a result of this study will be available for inspection on request by the Sponsor, the REC and representatives of the Human Tissue Authority.

PUBLICATION POLICY

The clinical study report will be used for publication and presentation at scientific meetings. Investigators have the right to publish orally or in writing the results of the study. Participants will not be identified in any publications.

Summaries of results will also be made available to Investigators for dissemination within their clinical areas (where appropriate and according to their discretion). At the end of the study, we will disseminate findings to the public and provide study information and updates on our website and newsletter and at public engagement events.

STUDY FINANCES

Funding source

This study is funded by Pfizer, Inc, University of Nottingham, NIHR Nottingham Biomedical Research Centre.

Participant stipends and payments

Participants will not be paid to participate in the study. Travel expenses will be offered for any hospital visits in excess of usual care.

SIGNATURE PAGES

Signatories to Protocol:

Chief Investigator: (name)____Guru Aithal__________

This protocol is confidential and the property of the University of Nottingham. No part of it may be transmitted, reproduced, published, or used by other persons without prior written authorisation from the University of Nottingham

Signature: 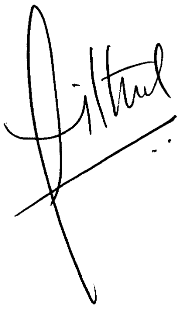

Date: ___________

Co- investigator: (name) _____Jane Grove_______________

Signature: 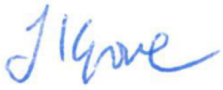

Date: ___________

Trial Statistician: (name)________Dingzhou Li______________________

Signature: 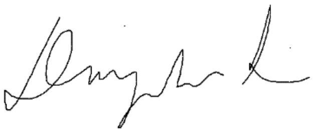

Date: ___________

REFERENCES

1. Topalian, S.L., et al., *Safety, activity, and immune correlates of anti-PD-1 antibody in cancer.* N Engl J Med, 2012. **366**(26): p. 2443-54.
2. Larkin, J., et al., *Combined Nivolumab and Ipilimumab or Monotherapy in Untreated Melanoma.* N Engl J Med, 2015. **373**(1): p. 23-34.
3. Haanen, J.B.A.G., et al., *Management of toxicities from immunotherapy: ESMO Clinical Practice Guidelines for diagnosis, treatment and follow-up†.* Annals of Oncology, 2017. **28**(suppl_4): p. iv119-iv142.
4. Puzanov, I., et al., *Managing toxicities associated with immune checkpoint inhibitors: consensus recommendations from the Society for Immunotherapy of Cancer (SITC) Toxicity Management Working Group.* Journal for immunotherapy of cancer, 2017. **5**(1): p. 95.
5. De Martin, E., et al., *Characterization of liver injury induced by cancer immunotherapy using immune checkpoint inhibitors.* J Hepatol, 2018. **68**(6): p. 1181-1190.

This protocol is confidential and the property of the University of Nottingham. No part of it may be transmitted, reproduced, published, or used by other persons without prior written authorisation from the University of Nottingham

6. Gauci, M.L., et al., *Immune-related hepatitis with immunotherapy: Are corticosteroids always needed?* J Hepatol, 2018. **69**(2): p. 548-550.
7. Affolter, T., et al., *Inhibition of immune checkpoints PD-1, CTLA-4, and IDO1 coordinately induces immune-mediated liver injury in mice.* PLoS One, 2019. **14**(5): p. e0217276.
8. Metushi, I.G., M.A. Hayes, and J. Uetrecht, *Treatment of PD-1(-/-) mice with amodiaquine and anti-CTLA4 leads to liver injury similar to idiosyncratic liver injury in patients.* Hepatology, 2015. **61**(4): p. 1332-42.
9. Zen, Y. and M.M. Yeh, *Hepatotoxicity of immune checkpoint inhibitors: a histology study of seven cases in comparison with autoimmune hepatitis and idiosyncratic drug-induced liver injury.* Modern Pathology, 2018. **31**(6): p. 965-973.
10. Roth, S.E., et al., *Next Generation DILI Biomarkers: Prioritization of Biomarkers for Qualification and Best Practices for Biospecimen Collection in Drug Development.* Clin Pharmacol Ther, 2019.
11. Church, R.J., et al., *Candidate biomarkers for the diagnosis and prognosis of drug-induced liver injury: An international collaborative effort.* Hepatology, 2019. **69**(2): p. 760-773.
12. Kaliyaperumal, K., et al., *Pharmacogenomics of drug-induced liver injury (DILI): Molecular biology to clinical applications.* J Hepatol, 2018. **69**(4): p. 948-957.
13. Nicoletti, P., et al., *Shared Genetic Risk Factors Across Carbamazepine-Induced Hypersensitivity Reactions.* Clin Pharmacol Ther, 2019.
14. Cirulli, E.T., et al., *A Missense Variant in PTPN22 is a Risk Factor for Drug-induced Liver Injury.* Gastroenterology, 2019. **156**(6): p. 1707-1716 e2.
15. Martens, A., et al., *Increases in Absolute Lymphocytes and Circulating CD4+ and CD8+ T Cells Are Associated with Positive Clinical Outcome of Melanoma Patients Treated with Ipilimumab.* Clin Cancer Res, 2016. **22**(19): p. 4848-4858.
16. Simeone, E., et al., *Immunological and biological changes during ipilimumab treatment and their potential correlation with clinical response and survival in patients with advanced melanoma.* Cancer Immunol Immunother, 2014. **63**(7): p. 675-83.
17. Martens, A., et al., *Baseline Peripheral Blood Biomarkers Associated with Clinical Outcome of Advanced Melanoma Patients Treated with Ipilimumab.* Clin Cancer Res, 2016. **22**(12): p. 2908-18.
18. Lou, Y., et al., *Epithelial-Mesenchymal Transition Is Associated with a Distinct Tumor Microenvironment Including Elevation of Inflammatory Signals and Multiple Immune Checkpoints in Lung Adenocarcinoma.* Clin Cancer Res, 2016. **22**(14): p. 3630-42.
19. Yamazaki, N., et al., *Cytokine biomarkers to predict antitumor responses to nivolumab suggested in a phase 2 study for advanced melanoma.* Cancer Sci, 2017. **108**(5): p. 1022-1031.
20. Fehrenbacher, L., et al., *Atezolizumab versus docetaxel for patients with previously treated non-small-cell lung cancer (POPLAR): a multicentre, open-label, phase 2 randomised controlled trial.* Lancet, 2016. **387**(10030): p. 1837-46.
21. Ji, R.R., et al., *An immune-active tumor microenvironment favors clinical response to ipilimumab.* Cancer Immunol Immunother, 2012. **61**(7): p. 1019-31.
22. Chifman, J., et al., *Conservation of immune gene signatures in solid tumors and prognostic implications.* BMC Cancer, 2016. **16**(1): p. 911.
23. Gentles, A.J., et al., *The prognostic landscape of genes and infiltrating immune cells across human cancers.* Nat Med, 2015. **21**(8): p. 938-945.
24. Tahara, H., et al., *Emerging concepts in biomarker discovery; the US-Japan Workshop on Immunological Molecular Markers in Oncology.* J Transl Med, 2009. **7**: p. 45.
25. Bedognetti, D., et al., *Gene-expression profiling in vaccine therapy and immunotherapy for cancer.* Expert Rev Vaccines, 2010. **9**(6): p. 555-65.
26. Ulloa-Montoya, F., et al., *Predictive gene signature in MAGE-A3 antigen-specific cancer immunotherapy.* J Clin Oncol, 2013. **31**(19): p. 2388-95.

This protocol is confidential and the property of the University of Nottingham. No part of it may be transmitted, reproduced, published, or used by other persons without prior written authorisation from the University of Nottingham

27. Page, D.B., et al., *Deep Sequencing of T-cell Receptor DNA as a Biomarker of Clonally Expanded TILs in Breast Cancer after Immunotherapy.* Cancer Immunol Res, 2016. **4**(10): p. 835-844.
28. Subudhi, S.K., et al., *Clonal expansion of CD8 T cells in the systemic circulation precedes development of ipilimumab-induced toxicities.* Proc Natl Acad Sci U S A, 2016. **113**(42): p. 11919-11924.
29. Tsujikawa, T., et al., *Quantitative Multiplex Immunohistochemistry Reveals Myeloid-Inflamed Tumor-Immune Complexity Associated with Poor Prognosis.* Cell Rep, 2017. **19**(1): p. 203-217.
30. Roh, W., et al., *Integrated molecular analysis of tumor biopsies on sequential CTLA-4 and PD-1 blockade reveals markers of response and resistance.* Sci Transl Med, 2017. **9**(379).
31. Donnem, T., et al., *Stromal CD8+ T-cell Density-A Promising Supplement to TNM Staging in Non-Small Cell Lung Cancer.* Clin Cancer Res, 2015. **21**(11): p. 2635-43.
32. Sato, K., et al., *Correlation between immune-related adverse events and efficacy in non-small cell lung cancer treated with nivolumab.* Lung Cancer, 2018. **115**: p. 71-74.
33. Shahabi, V., et al., *Gene expression profiling of whole blood in ipilimumab-treated patients for identification of potential biomarkers of immune-related gastrointestinal adverse events.* J Transl Med, 2013. **11**: p. 75.
34. Ji, C., et al., *Myocarditis in Cynomolgus Monkeys Following Treatment with Immune Checkpoint Inhibitors.* Clin Cancer Res, 2019. **25**(15): p. 4735-4748.
35. Antoine, D.J., et al., *Mechanistic biomarkers provide early and sensitive detection of acetaminophen-induced acute liver injury at first presentation to hospital.* Hepatology, 2013. **58**(2): p. 777-87.
36. Foureau, D.M., et al., *Comparative analysis of portal hepatic infiltrating leucocytes in acute drug-induced liver injury, idiopathic autoimmune and viral hepatitis.* Clin Exp Immunol, 2015. **180**(1): p. 40-51.
37. Taubert, R., et al., *Intrahepatic regulatory T cells in autoimmune hepatitis are associated with treatment response and depleted with current therapies.* J Hepatol, 2014. **61**(5): p. 1106-14.
38. Benesic, A., A. Leitl, and A.L. Gerbes, *Monocyte-derived hepatocyte-like cells for causality assessment of idiosyncratic drug-induced liver injury.* Gut, 2016. **65**(9): p. 1555-1563.
39. Russo, M.W., et al., *Profiles of miRNAs in serum in severe acute drug induced liver injury and their prognostic significance.* Liver Int, 2017. **37**(5): p. 757-764.
40. Aithal, G.P., et al., *Case definition and phenotype standardization in drug-induced liver injury.* Clin Pharmacol Ther, 2011. **89**(6): p. 806-15.

Appendix 1. Patient assessment schedule

Controls	**Before starting CPI**	**After 6-14 weeks of treatment**	
Cases	**Day 0 (date of reaction)**	**Day 7 (2-14 after Visit 1)**	**Day 30 ±7**
	**Visit 1**	**Visit 2**	**Visit 3**
Inclusion/Exclusion criteria	X		
Medical history/current medical condition	X		

This protocol is confidential and the property of the University of Nottingham. No part of it may be transmitted, reproduced, published, or used by other persons without prior written authorisation from the University of Nottingham

Demography	X		
Body height	X		
Body weight	X		
Drug history in past 6 months, including OTC, herbal, dietary supplement, recreational drugs	X		
Smoking history	X		
Alcohol, quantitative assessment	X		
Viral hepatitis screen	X		
Autoantibody* screening	X		(X)**
Liver ultrasound	X		
Standard clinical analysis: Hematology, Blood chemistry, (Urine analysis)	X	X	X
Blood sample collection	X	X	X
Urine sample collection if possible	X	X	X
Stool sample collection if possible	X	X	X
Study completion information			X

*Autoantibodies: antinuclear (ANA), anti-smooth muscle (ASMA), antimitochondrial (AMA) and liver kidney microsomal type 1 (LKM-1)
**Repeat autoantibody screening if positive in week 1

This protocol is confidential and the property of the University of Nottingham. No part of it may be transmitted, reproduced, published, or used by other persons without prior written authorisation from the University of Nottingham
