# Supplementary material for: Circulating exhausted CD8+ effector memory cells differentiate immune checkpoint inhibitor-induced liver injury from other acute immune-mediated liver injuries
Source: J Immunother Cancer. 2026 Mar 27;14(3):e014178. doi: 10.1136/jitc-2025-014178 (PMC13034233; doi:10.1136/jitc-2025-014178)
Supplement: online supplemental file 2 [file jitc-14-3-s002.docx]

**Circulating exhausted CD8+ effector memory cells differentiate immune checkpoint inhibitor-induced liver injury from other acute immune-mediated liver injuries**

Stuart Astbury, Edmond Atallah, Jane I Grove, Amber G Bozward, Scott P Davies, Mark J Sheehan, Steven W Kumpf, Jessie Qian, Natalia M Krajewska, Grace E Wootton, Melanie R Lingaya, Davor Kresnik, Flavia Radulescu, Ankit Rao, Hester Franks, Lourdes Ruiz-Ortega, Mar Riveiro-Barciela, Shashi K Ramaiah, Thomas A Lanz, Changhua Ji, Poulam M Patel, Ye H Oo & Guruprasad P Aithal

**Supplementary material**

Table of Contents

[Supplementary figures 2](#_Toc212887997)

[Supplementary tables 10](#_Toc212887998)

[Supplementary methods 16](#_Toc212887999)

[Patient recruitment 16](#_Toc212888000)

[Statistical analysis 17](#_Toc212888001)

[Mass cytometry 17](#_Toc212888002)

[Flow cytometry 20](#_Toc212888003)

[scRNA-seq 21](#_Toc212888004)

[Bulk RNAseq 22](#_Toc212888005)

[Cytokine profiling 23](#_Toc212888006)

[Immunofluorescence 24](#_Toc212888007)

[Code availability 25](#_Toc212888008)

[References 26](#_Toc212888009)

## Supplementary figures

**
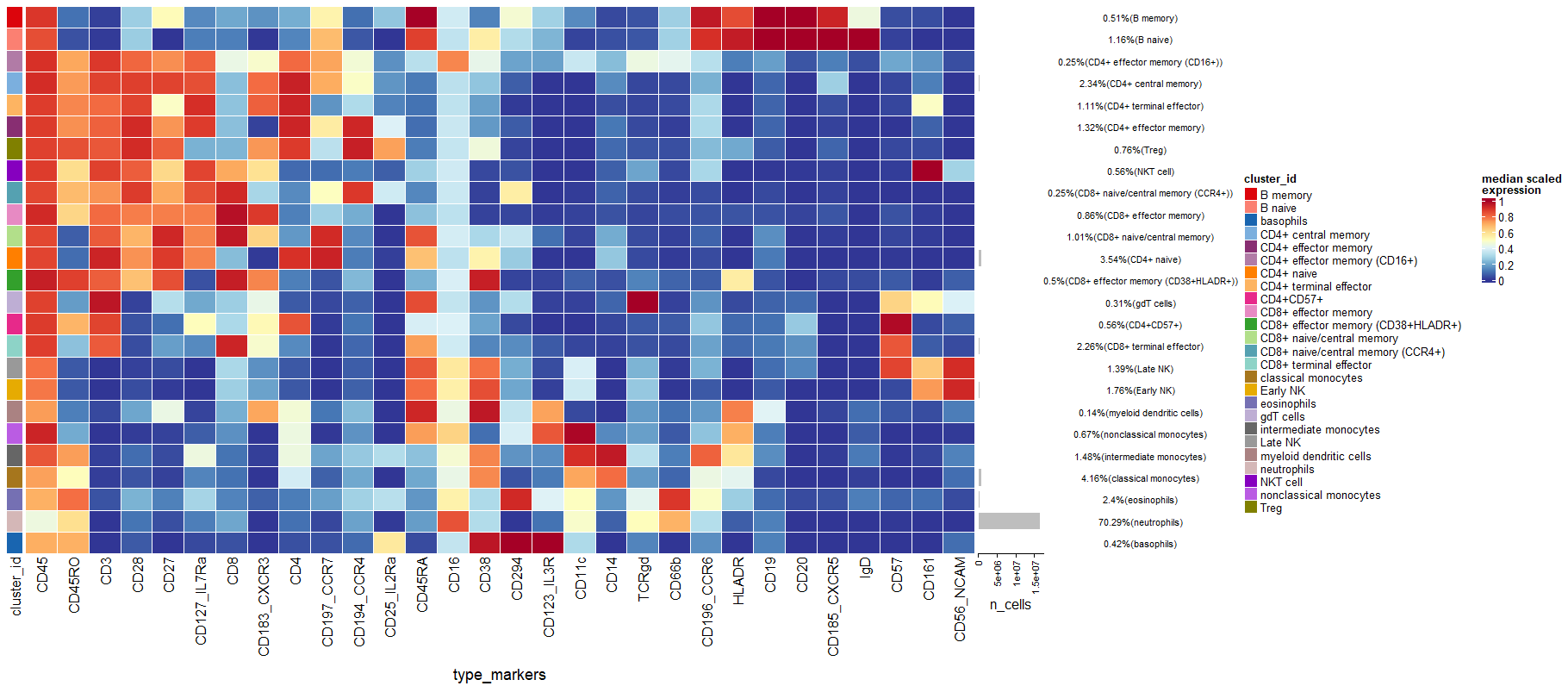
**

**Supplementary figure 1**: Heatmap summarising marker expression (x axis) for each annotated cluster (y axis) for all cells (23,451,791).

**A**

**
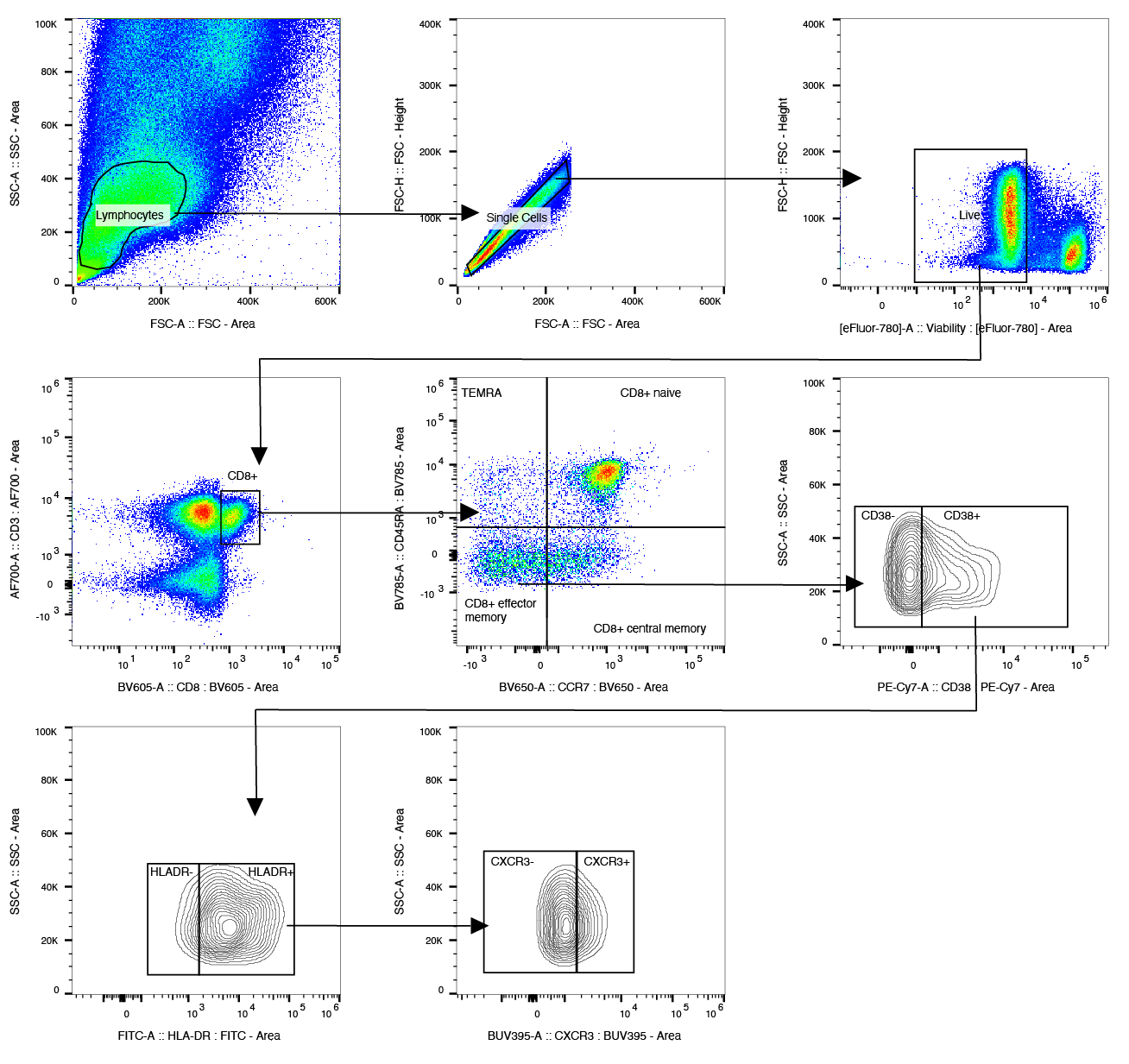
**

**B**


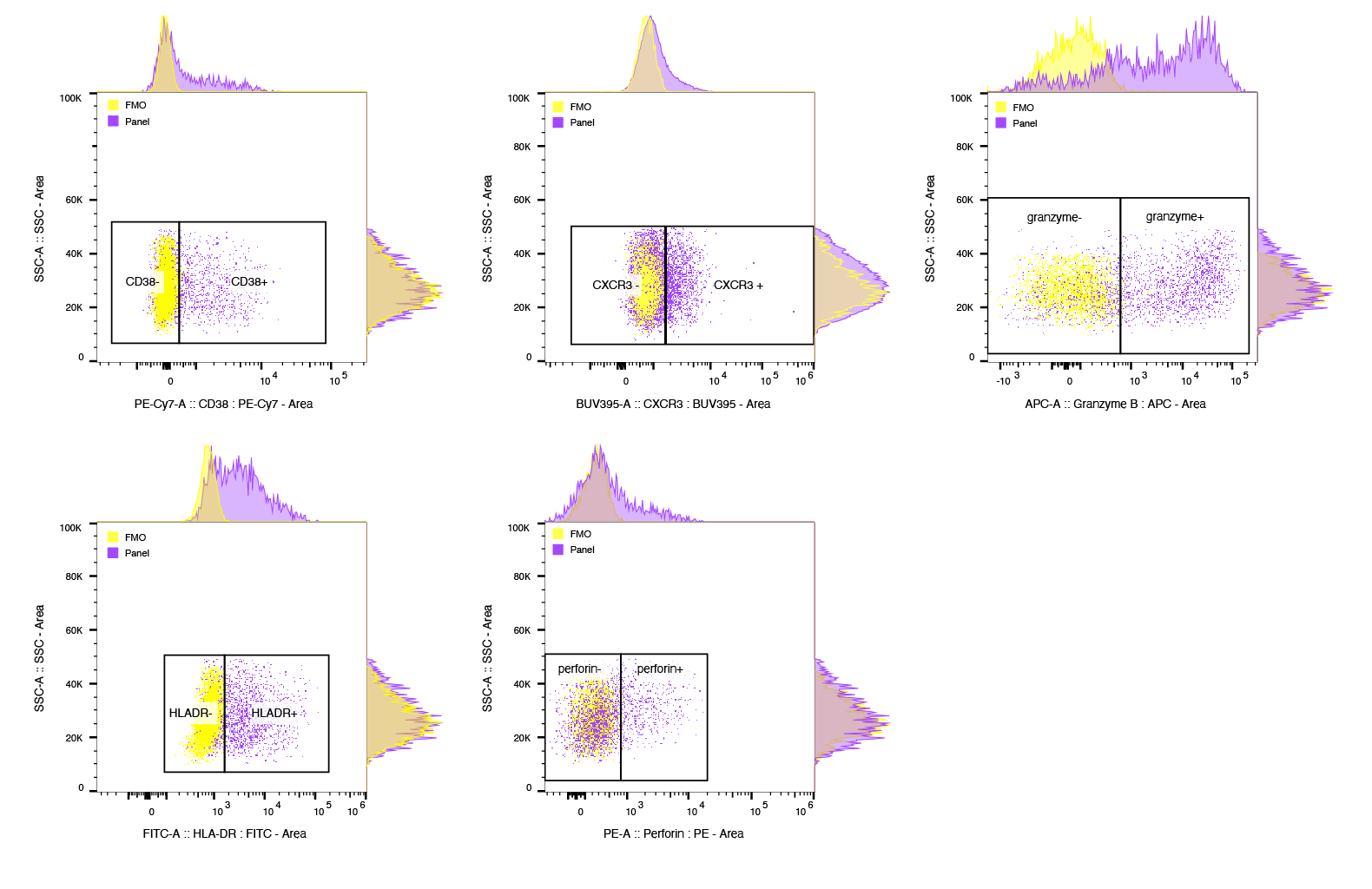


**Supplementary figure 2:** **A**: Gating strategy for live CD8^+^ subsets using CD8, CD3, CD45RA and CCR7 antibodies. **B:** Fluorescence minus one (FMO) controls for gating of CXCR3, CD38, HLA-DR, granzyme and perforin.


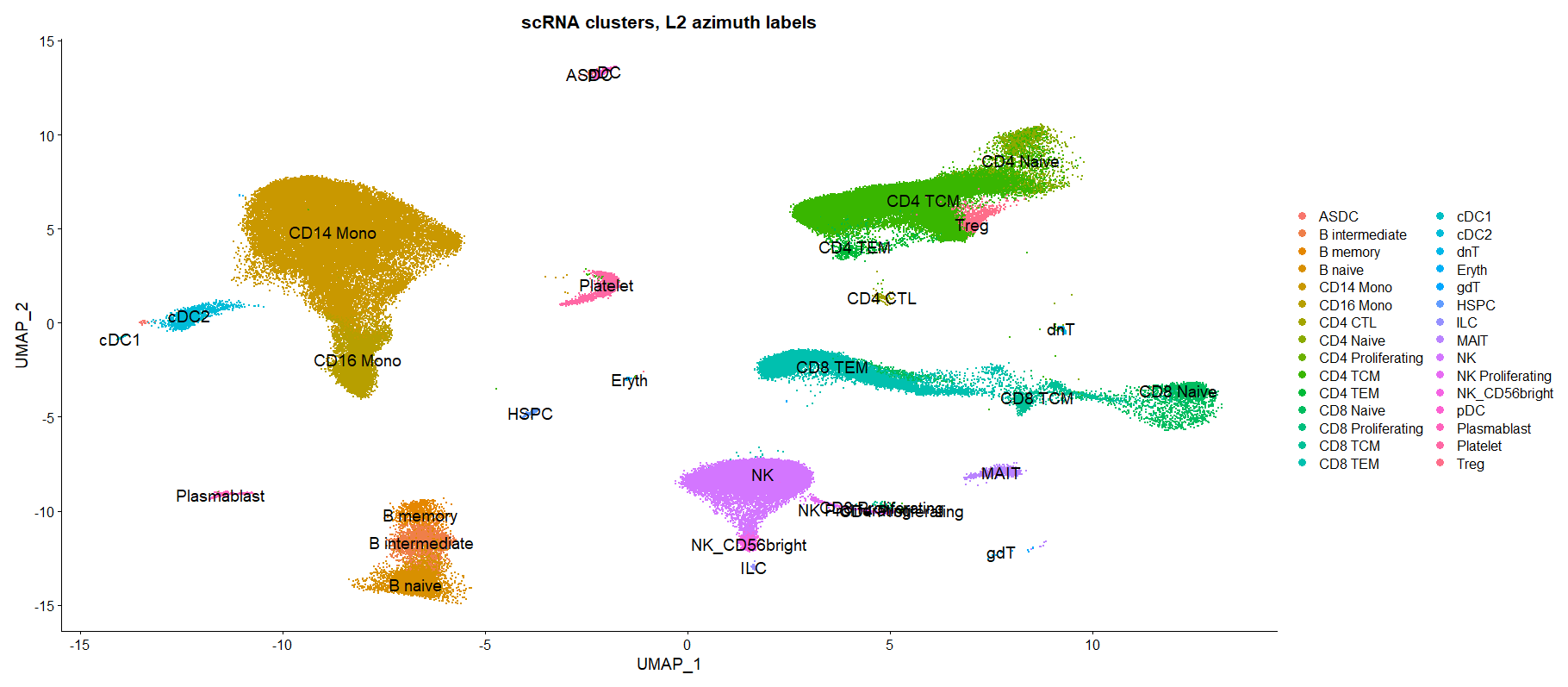


**Supplementary figure 3**: Uniform manifold and approximation projection (UMAP) plot of single cell RNA sequencing data from PBMC samples. Cell clusters are labelled using Azimuth level 2 labels from the NIH Human Biomolecular Atlas PBMC reference.


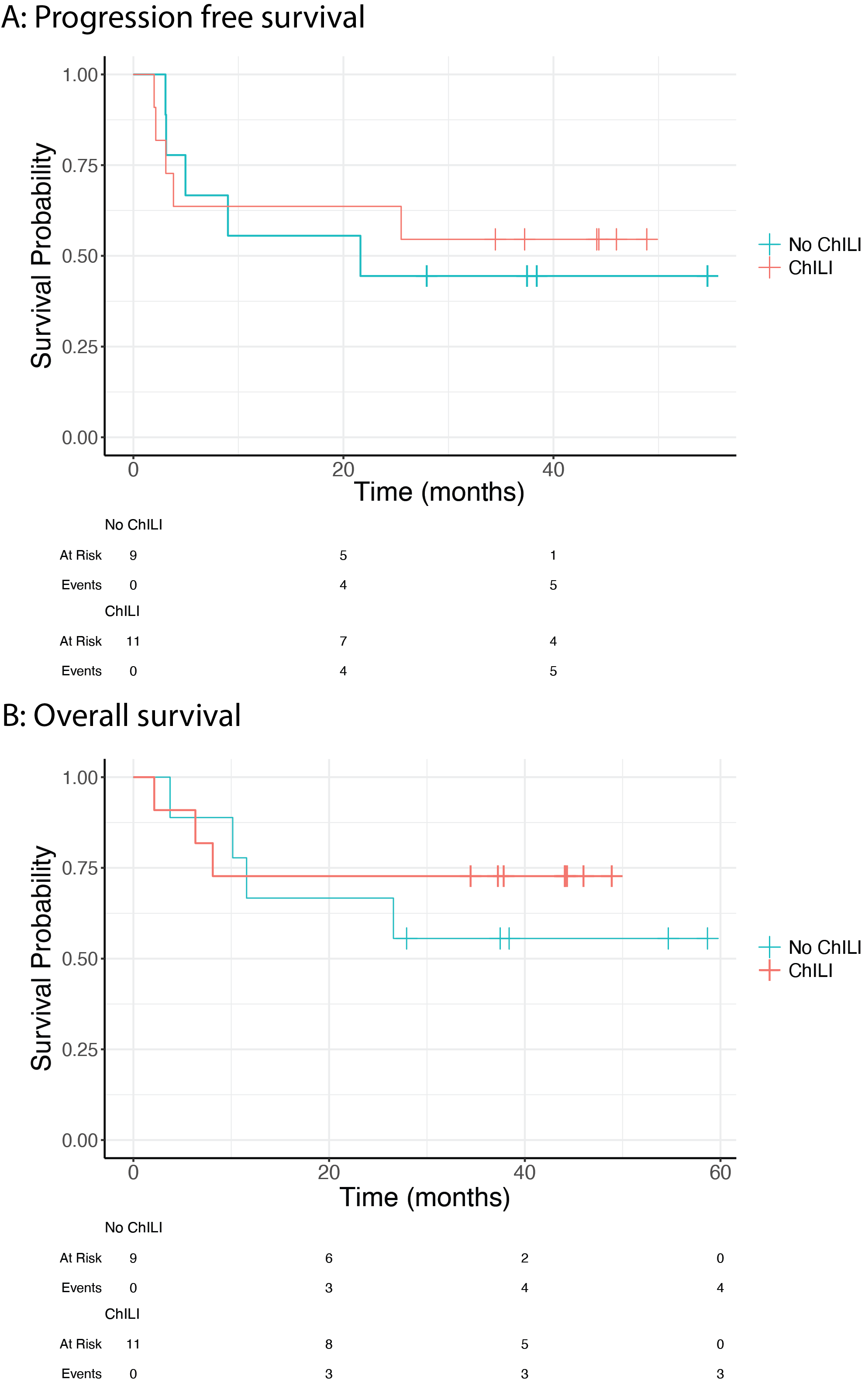


**Supplementary figure 4:** Progression free (A) and overall (B) survival comparing patients in the study with and without ChILI. To compare the largest homogenous groups this analysis was restricted to patients with metastatic melanoma receiving combination Ipilimumab/Nivolumab CPI therapy only. No ChILI n=9, ChILI n=11.

**
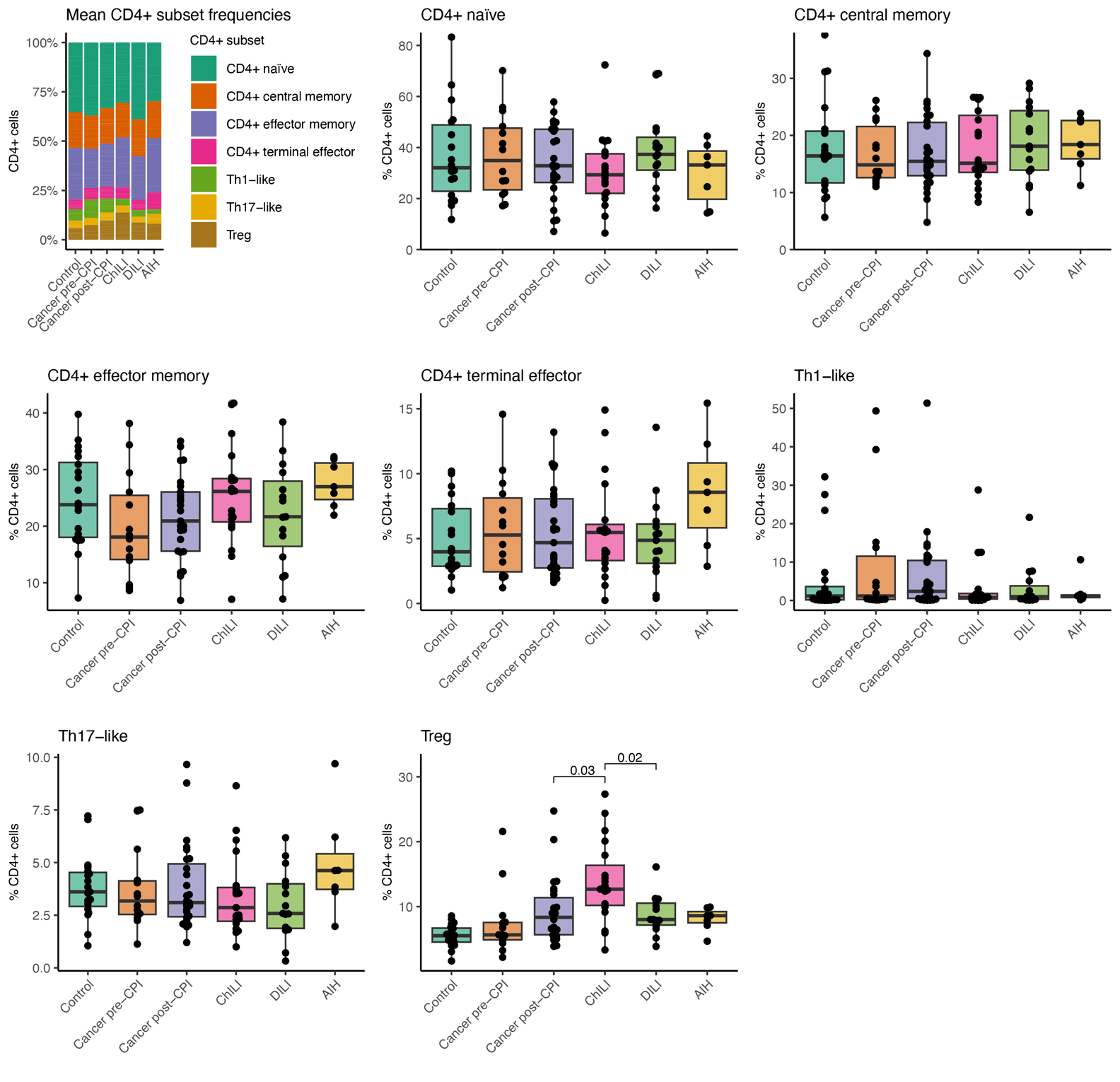
**

**Supplementary figure 5**: Percentage abundance of CD4^+^ clusters identified in mass cytometry data using FlowSOM. Control n=15, Cancer pre-CPI n=14, Cancer post-CPI n=21 ChILI n=16, DILI n=13, AIH n=6. CD4^+^ clusters were defined as CD4^+^CD3^+^CD8^-^CD66b^-^ CD14^-^ TCRgd^-^CD11c^-^. CD4^+^ subsets were defined as naïve (CD45RA^+^CD27^+^CD28^+^CCR7^+^CD45RO^-^), central memory (CD45RA^low^CD27^+^CD28^+^CCR7^mid^CD45RO^+^), effector memory (CD45RA^low^CD27^+^CD28^+^CCR7^low^CD45RO^+^), terminal effector (CD45RA^low^CD27^-^CD28^+^CCR7^-^CD45RO^+^), Th1-like (CXCR3^+^CCR6^-^CXCR5^-^), Th17-like (CXCR3^-^CCR6^+^CXCR5^-^CCR4^+^) and Treg (CD25^+^CCR4^+^CD127^-^). Th2-like cells (CXCR3^-^CCR6^-^CXCR5^-^CCR4^+^) were not present in sufficient numbers to be defined as a distinct cluster. Pairwise comparisons were made between clusters using the R package diffcyt.


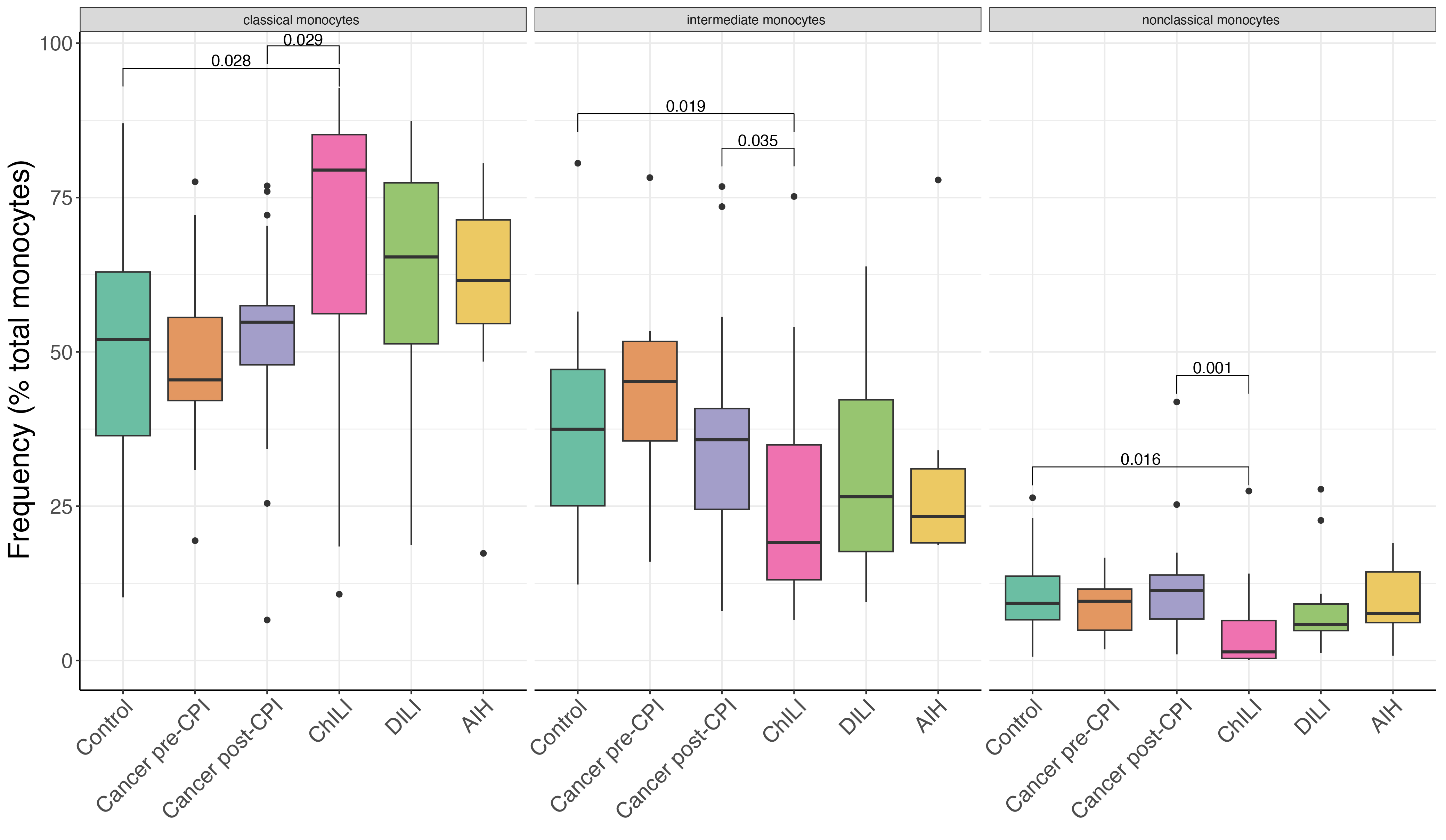


**Supplementary figure 6:** Monocyte subsets identified using FlowSOM and compared between experimental groups. Control n=15, Cancer pre-CPI n=14, Cancer post-CPI n=21 ChILI n=16, DILI n=13, AIH n=6. Monocyte clusters were first defined as CD3-CD19-CD56-CD66b-HLA-DR+CD11c+ and subsets defined as classical (CD14^+^CD16^-^HLA-DR^+^CD38^+^), intermediate (CD14^+^CD16^low^HLA-DR^high^CD38^low^) and nonclassical (CD14^-^CD16^+^HLA-DR^+^CD38^-^). Pairwise comparisons were made between clusters using the R package diffcyt.


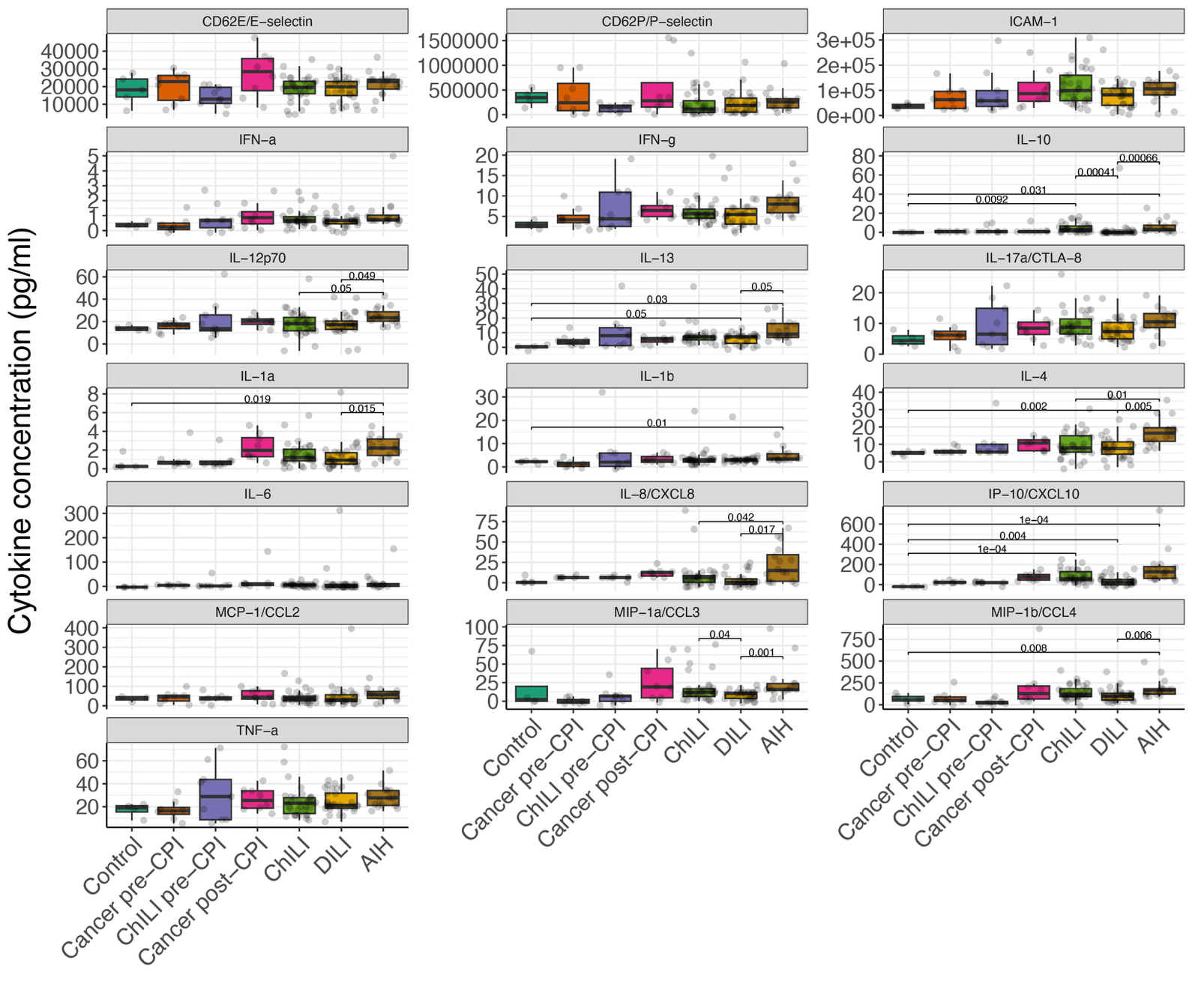
**Supplementary figure 7**: Plasma cytokines in Control (n=5), Cancer pre-CPI (n=8), ChILI pre-CPI (n=9), Cancer post-CPI (n=8), ChILI (n=34), DILI (n=34) and AIH (n=16) samples. Comparisons between groups were performed using Mann-Whitney U test and adjusted for multiple comparisons using the Benjamini-Hochberg procedure.


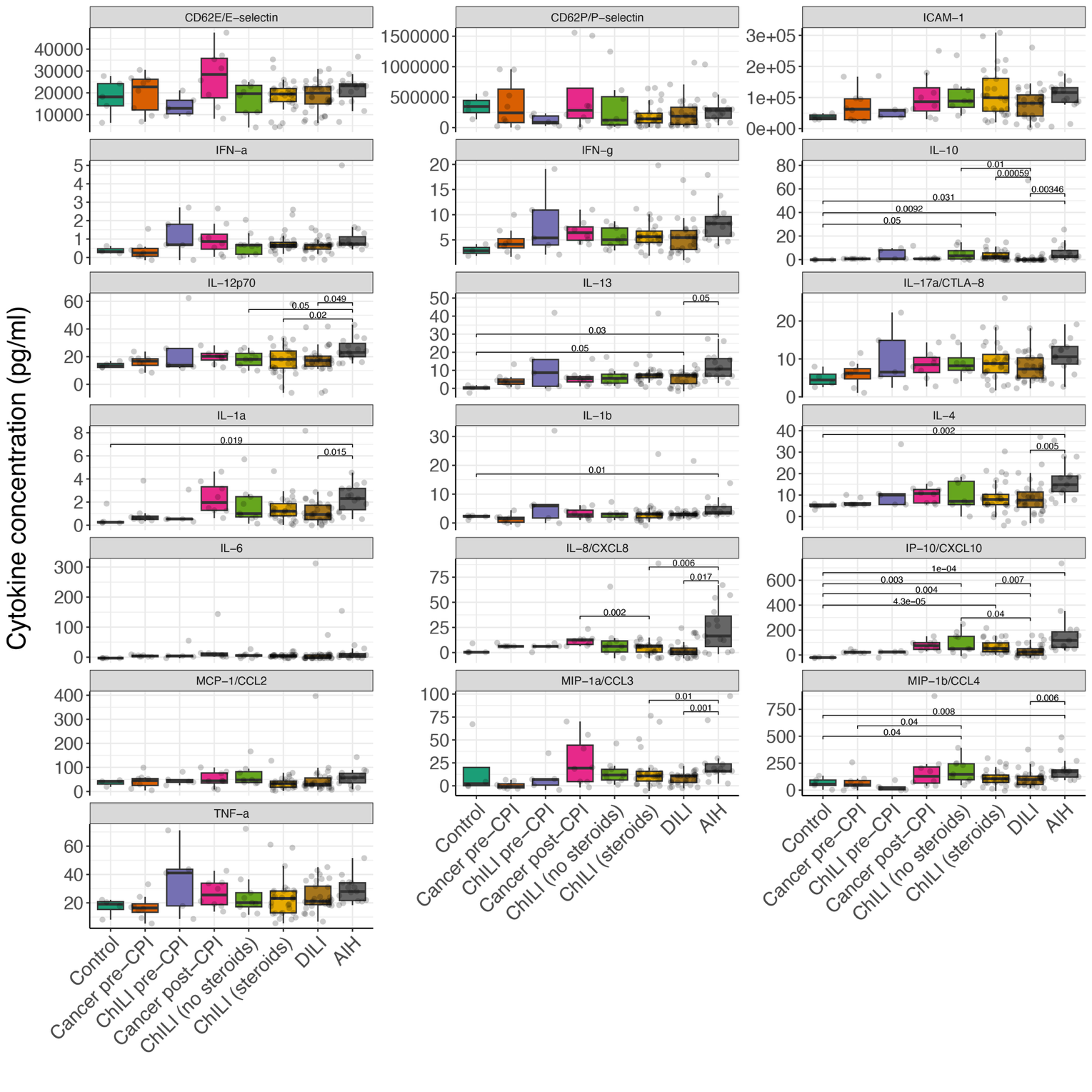


**Supplementary figure 8**: Plasma cytokines with ChILI group split into patients who did and did not receive steroids before sampling. Control (n=5), Cancer pre-CPI (n=8), ChILI pre-CPI (n=9), Cancer post-CPI (n=8), ChILI (no steroids) (n=9), ChILI (steroids) (n=30), DILI (n=34) and AIH (n=16). Comparisons between groups were performed using Mann-Whitney U test and adjusted for multiple comparisons using the Benjamini-Hochberg procedure.

**Supplementary figure 9**: Percentage abundance of the CD38^+^HLA-DR^+^CXCR3^+^ CD8^+^ subset displayed in Figure 1, with Cancer post-CPI patients who developed other immune-related adverse events based on ESMO guidelines (n=6, 3 colitis, 1 pancreas, 1 skin, thyroid and nervous system, 1 skin, colitis and adrenal).

## Supplementary tables

**Supplementary table 1:** Clinical characteristics and liver enzymes of patients with liver biopsies taken for bulk RNAseq.

|  | **ChILI (n=16)** | **DILI (n=10)** | **AIH (n=10)** |
| --- | --- | --- | --- |
| **Mean age, years (SD)** | 70 (11) | 61 (13) | 61 (16) |
| **Sex, n (Male, %)** | 8 (50) | 2 (20) | 2 (20) |
| **Type of cancer** | Malignant melanoma **(n=5)**  Non-small cell lung cancer **(n=5)**  Renal cell carcinoma **(n=1)**  Duodenal adenocarcinoma **(n=1)**  Rectal adenocarcinoma **(n=1)**  Sigmoid adenocarcinoma **(n=1)**  Breast cancer **(n=1)** | NA **(n=5)**  Breast cancer **(n=2)**  Ovarian cancer **(n=1)**  Renal cell carcinoma **(n=1)**  Oral cancer **(n=1)** | N/A |
| **CPI regime/class of causative drug** | Combination anti-CTLA-4/anti-PD-1 **(n=5)**  Anti PD-1 **(n=8)**  Anti PD-L1 **(n=3)** | Tyrosine kinase inhibitor **(n=4)**  Herbal medicine **(n=2)**  Non-steroidal anti-inflammatory **(n=1)**  Histone deacetylase inhibitor **(n=1)**  Antibiotic **(n=1)**  Disulfiram **(n=1)** | N/A |
| **Patients received corticosteroids prior to sampling, n (%)** | 0 (0) | 2 (20) | 0 |
| **ALT, median IU (IQR)** | 624 (500, 842) | 621 (392, 1341) | 464 (289, 792) |
| **ALP, IU, median IU (IQR)** | 161 (128, 452) | 224 (123, 283) | 146 (110, 179) |
| **Total bilirubin, median μmol/L (IQR)** | 16 (14, 37) | 47 (28, 96) | 38 (15, 153) |

**Supplementary table 2**: Mass cytometry antibodies and isotope conjugates from Maxpar Direct Immune Profiling kit

| **Antibody (clone)** | **Isotope** |
| --- | --- |
| CD45 (HI30) | ^89^Y |
| CD196/CCR6 (G034E3) | ^141^Pr |
| CD123 (6H6) | ^143^Nd |
| CD19 (HIB19) | ^144^Nd |
| CD4 (RPA-T4) | ^145^Nd |
| CD8a (RPA-T8) | ^146^Nd |
| CD11c (Bu15) | ^147^Sm |
| CD16 (3G8) | ^148^Nd |
| CD45RO (UCHL1) | ^149^Sm |
| CD45RA (HI100) | ^150^Nd |
| CD161 (HP-3G10) | ^151^Eu |
| CD194/CCR4 (L291H4) | ^152^Sm |
| CD25 (BC96) | ^153^Eu |
| CD27 (O323) | ^154^Sm |
| CD57 (HCD57) | ^155^Gd |
| CD183/CXCR3 (G025H7) | ^156^Gd |
| CD185/CXCR5 (J25D4) | ^158^Gd |
| CD28 (CD28.2) | ^160^Gd |
| CD38 (HB-7) | ^161^Dy |
| CD56/NCAM (NCAM16.2) | ^163^Dy |
| TCRgd (B1) | ^164^Dy |
| CD294 (BM16) | ^166^Er |
| CD197/CCR7 (G043H7) | ^167^Er |
| CD14 (63D3) | ^168^Er |
| CD3 (UCHT1) | ^170^Er |
| CD20 (2H7) | ^171^Yb |
| CD66b (G10F5) | ^172^Yb |
| HLA-DR (LN3) | ^173^Yb |
| IgD (IA6-2) | ^174^Yb |
| CD127 (A019D5) | ^176^Yb |
| Cell-ID Intercalator-103Rh | ^103^Rh |

**Supplementary table 3:** Flow cytometry antibodies and fluorophores

| **Antibody (clone)** | **Fluorophore** | **Manufacturer and catalogue number** | **Isotype (manufacturer and catalogue number)** |
| --- | --- | --- | --- |
| HLA-DR (L243) | FITC | Biolegend (307604) | Anti-IgG2a κ MOPC-173 (Biolegend 400208) |
| CTLA-4 (L3D10) | PE/Dazzle | Biolegend (349921) | Anti-IgG2a κ MOPC-173 (Biolegend 400275) |
| Granzyme B (QA16A02) | APC | Biolegend (372203) | Anti-IgG1 κ MOPC-21 (BDBioscience 550854) |
| CD3 (UCHT1) | AF700 | Biolegend (300424) | Anti-IgG1 κ MOPC-21 (Biolegend 400143) |
| PD-1 (EH12.2H7) | BV421 | Biolegend (329920) | Anti-IgG1 κ MOPC-21 (Biolegend 400154) |
| CD8 (SK1) | BV605 | Biolegend (344741) | Anti-IgG1 κ MOPC-21 (Biolegend 400161) |
| CCR7 (3D12) | BV650 | BDBioscience (563407) | Anti-IgG2a κ MOPC-173 (Biolegend 400265) |
| CD45RA (HI100) | BV785 | BDBioscience (563870) | Anti-IgG2b κ MOPC-21 (Biolegend 400169) |
| CD38 (HIT2) | PE-Cy7 | Invitrogen (25-0389-42) | Anti-IgG1 κ MOPC-21 (BDBioscience 557872) |
| Perforin (dG9) | PE | eBioscience (12-9994-42) | Anti-IgG1 κ MOPC-21 (Biolegend 400111) |
| CXCR3 (1C6) | BUV395 | BDBioscience (565223) | Anti-IgG1 κ X40 (BDBioscience 563547) |
| Viability marker | e-Fluor 780 | Invitrogen (65-0865-14) | NA |

**Supplementary table 4:** Immunofluorescence microscopy antibodies

| **Antibody (clone)** | **Manufacturer and catalogue number** | **Secondary and fluorophore** | **Manufacturer and catalogue number** |
| --- | --- | --- | --- |
| E-cadherin (36) | BD Biosciences (610182) | Goat anti-mouse IgG2a AF546 | Invitrogen (**A-21133)** |
| CD38 (EPR4106) | Abcam (ab108403) | Horse anti-rabbit DyLight594 | Vector Laboratories/2B Scientific (DI-1794-15) |
| CXCR3 (5C10E6) | Novus Biologicals (NBP2-61681) | Goat anti-mouse IgG1 AF488 | Invitrogen (A-11001) |
| CD8 (4B11) | Invitrogen (MA1-80231) | Goat anti-mouse IgG2b AF647 | Invitrogen (A-21235) |

## Supplementary methods

### Patient recruitment

Patients identified through prospective monitoring of treatment with CPI and/ or referred with acute manifestations of liver injury were prospectively recruited and sampled at the time of liver injury at NUH. In addition, healthy volunteers were enrolled. Studies were ethically approved by North West – Haydock, Yorkshire & The Humber – Leeds East and NHS Wales Research Ethics Committees (approvals 20/NW/0274, 15/YH/0294 and 18/WA/0214 respectively). Liver biopsies from steroid naïve patients were obtained with ethical approval granted by the Vall d’Hebron Hospital ethics committee and Spanish Agency of Medicines and Medical Devices (reference PR(AG)481/2018). All participants gave written informed consent.

All patients with ChILI, DILI and AIH met the biochemical criteria as defined previously [1], having serum ALT ≥ 5x ULN or ALT ≥ 3x ULN plus TBL ≥ 2x ULN or ALP ≥ 2x ULN (with accompanying elevations of gamma-glutamyl transferase) at the time of enrolment. ChILI and DILI were diagnosed based on the presence of a compatible temporal sequence between drug intake and detection of liver injury as well as test results to exclude alternative conditions, which included, but were not limited to, viral serology, viral load, imaging tests, presence of autoantibodies, immunoglobulin G values and biopsy findings (when available). AIH was diagnosed based on the guidelines from the American Association for the Study of Liver Diseases [2]. All patients in the AIH group had undergone liver biopsy as a part of their investigations and samples were obtained prior to introducing corticosteroid therapy.

### Statistical analysis

Demographic and clinical data are described using mean ± standard deviation (SD) for continuous measurements that are normally distributed, median and inter-quantile range (IQR) for non-normally distributed continuous variables and frequencies and percentiles for categorical data. Patients' pathological and clinical characteristics were compared using the Chi-square test for categorical variables or Fisher's exact test when one or more expected cell counts were less than 5. For continuous variables, Student's t-test was applied. For continuous outcome variables exhibiting a skewed distribution, Kruskal-Wallis H test was used for independent variables and Wilcoxon signed-rank test for paired data.

### Mass cytometry

#### Sample preparation

Whole blood samples were collected in sodium heparin vacutainer tubes and were prepared according to the Maxpar Direct Immune Profiling kit (Standard BioTools) protocol. 270μL whole blood was blocked with sodium heparin (Sigma-Aldrich, 10μL per 1mL whole blood at 10KU/mL) before incubation with the lyophilised antibodies (antibodies and conjugates are included in supplementary table 2). Red blood cells were lysed (Cal-Lyse, Thermo Fisher) and removed through washing with sterile water and Maxpar cell staining buffer, the remaining cells were fixed in 1.6% formaldehyde. Cells were incubated in Maxpar Fix and Perm buffer (containing a DNA intercalator conjugated to ^191^Ir and ^193^Ir) overnight at 4°C before freezing and storing at -80°C. For running samples were thawed gently and washed in Maxpar cell acquisition solution before running on a Helios mass cytometer (Standard BioTools). A maximum of 300,000 events were acquired for each sample.

#### Cleanup and QC

R version 4.2.2 was used for all processing. Normalised data were exported from the Helios in FCS format and the R package CyTOFclean (v1.0.3) was used to gate on gaussian parameters (residual, offset, center and width) and event length to exclude doublets and other artifacts, and gating on the isotopes ^140^Ce, ^142^Ce, ^165^Ho and ^175^Lu was used to remove normalisation beads. Gating on ^191^Ir and ^193^Ir was carried out manually using FlowJo v10 software (FlowJo LLC) to exclude dead cells.

#### Clustering

Clustering was carried out according to the CyTOF workflow [3]. Cleaned event data for each sample were arcsinh transformed with a cofactor of 5 and imported into the R package CATALYST v1.18.1. To obtain a coefficient of variation of 5% in clusters of 1% abundance, samples with fewer than 40,000 acquired events post-quality control were excluded, numbers of excluded samples are detailed in Figure 1. Cell clusters were determined with the R package FlowSOM v2.2.0 [4] using a 10x10 self-organising map, these 100 nodes were then summarised to 42 clusters using ConsensusClusterPlus v1.58.0 [5]. 42 clusters were selected to enable detection of the 37 cell types according to the Maxpar Direct Immune Profiling kit, with headroom to detect clusters unique to experimental groups. Clustering was repeated using 50 different random starts to ensure reproducibility. Clusters were manually annotated, those containing non-immune cell types (CD45^-^) or making up <1% of all cells across all samples were excluded from further analyses. A heatmap illustrating marker expression for each cluster is included as supplementary figure 2.

Once identified in the initial clustering, CD4 and CD8 clusters were extracted and re-clustered using only relevant markers to identify T-cell subsets. CD4^+^ cells were defined as CD4^+^CD3^+^CD8^-^CD66b^-^ CD14^-^ TCRgd^-^CD11c^-^, and staged using the following markers: naïve (CD45RA^+^CD27^+^CD28^+^CCR7^+^CD45RO^-^), central memory (CD45RA^low^CD27^+^CD28^+^CCR7^mid^CD45RO^+^), effector memory (CD45RA^low^CD27^+^CD28^+^CCR7^low^CD45RO^+^), terminal effector (CD45RA^low^CD27^-^CD28^+^CCR7^-^CD45RO^+^), Th1-like (CXCR3^+^CCR6^-^CXCR5^-^), Th17-like (CXCR3^-^CCR6^+^CXCR5^-^CCR4^+^) and Treg (CD25^+^CCR4^+^CD127^-^). Th2-like cells were defined as CXCR3^-^CCR6^-^CXCR5^-^CCR4^+^ but due to low abundance were not visible as a distinct cluster via FlowSOM.

CD8^+^ cells were defined as CD8^+^CD3^+^CD66b^-^CD19^-^CD4^-^CD14^-^CD161^-^TCRgd^-^CD123^-^CD11c^-^ and staged using the following markers: naïve/central memory (CD27^high^CD45RA^high^CD28^mid^CCR7^high^CD45RO^low^), effector memory (CD27^mid^CD45RA^low^CD28^mid^CCR7^low^CD45RO^mid^) and terminal effector (CD27^-^CD45RA^high^CD28^low^CCR7^-^CD45RO^low^).

Differential expression was determined using the R package diffcyt v1.14.0 [6], making pairwise comparisons between experimental groups. All reported p values are adjusted for multiple comparisons using the Benjamini-Hochberg method.

#### Pseudotime analysis

To determine relative levels of differentiation and branching cell trajectories, CD8^+^ clusters were extracted for pseudotime analysis using the R package Slingshot v2.2.1 [7]. CD8^+^ clusters were extracted from the dataset, and markers relevant to CD8^+^ T-cell differentiation (CD45RA, CD45RO, CD27, CD28, CCR7, CD38, CXCR3 and HLA-DR) were used to calculate pseudotime trajectories. Finally, these markers were used to calculate a pseudotime value for each cell in its respective lineage, with the Naïve/central memory CD8^+^ cluster defined as the starting point. Trajectories were then plotted on a uniform manifold approximation and projection (UMAP) plot, using a random sample of 100,000 cells for plot legibility.

### Flow cytometry

Concurrently with samples taken for mass cytometry, PBMC were isolated within 1hr of blood draws using Ficoll separation. PBMC were frozen in ABC Media (CTL-Cryo) and stored in liquid nitrogen. For flow cytometry PBMC were thawed in RPMI before counting, washing in PBS and normalising to 1 million cells/mL. Cells were suspended in flow cytometry staining buffer (Invitrogen) before addition of surface markers (CD3, CD8, HLA-DR, PD-1, CCR7, CD45RA, CD38, CXCR3) and incubation for 30 minutes at 4°C. Cells were incubated with Cytofix (Invitrogen) for 45 minutes at 4°C before washes with permeabilisation buffer (Invitrogen). Following washing intracellular markers (granzyme B, perforin, CTLA-4) were added and cells were incubated at 4°C for 30 minutes. Following final washes cells were suspended in Cytofix before analysis on a ID7000 spectral flow cytometer (Sony Biotechnology). Antibodies and matched isotype controls used for flow cytometry are included in supplementary table 3.

CD8^+^ cells were identified by gating on CD3 and CD8, and CD8^+^ and subpopulations were defined by CCR7 and CD45RA expression as naïve (CCR7^+^CD45RA^+^), central memory (CCR7^+^CD45RA^-^), effector memory (CCR7^-^CD45RA^-^), and effector memory re-expressing CD45RA (TEMRA, CCR7^-^CD45RA^+^), outlined in supplementary figure 1A. The effector memory population of interest was identified by gating on CD38, HLA-DR and CXCR3 (supplementary figure 1B). Gates were placed using fluorescence minus one (FMO) controls. All analyses were carried out in FlowJo software v10 (FlowJo LLC).

### scRNA-seq

scRNA-seq was performed using the 10x Genomics single-cell 5′ Gene Expression and V(D)J library platform. PBMC were drop-wise thawed per the 10x Genomics thawing guidelines. Dead cells were then removed using the Dead Cell Removal Kit (Miltenyi Biotec) and remaining cells were stained and counted using the ReadyProbes Cell Viability Imaging Kit (Thermo Fisher Scientific) on the Countess 3 Automated Cell Counter. The concentration of single-cell suspensions was targeted to 1200 live cells/μl. Cells were loaded for targeted recovery of 10,000 cells/chip position. Single-cell libraries were generated with Chromium Single Cell 5’ Gene Expression and V(D)J Reagent Kit (10x Genomics) per the manufacturer’s instruction. Purified libraries were sequenced on the Illumina NextSeq 2000 with 200 cycle kits. Matched PBMC samples were used for single cell RNA sequencing in a subset of patients (cancer pre-CPI, cancer post-CPI and ChILI, 10 patients per group). Sequence reads were demultiplexed and QCd using CellRanger (10x Genomics) and processed using the R package Seurat (v5.3.0). Following filtering on UMI count (removing cells <200 and >2500 counts to exclude dead cells and doublets respectively) and cells with mitochondrial DNA >5% an average of 3940 cells/sample were used, with 2693 reads/cell. Cell clusters were annotated using the human PBMC reference (HuBMAP Consortium) [8], before CD8+ clusters were extracted and re-clustered using the default Louvain algorithm in Seurat. Differential abundance testing of clusters was carried out using the R package speckle (v1.8.0), first applying a variance stabilising logit transform to the cluster proportions for each sample, then running a linear model via the package limma (v3.64.1). Potential transcription factors regulating the CD38+HLA-DR+CXCR3+ cluster of interest were assessed by first using the FindMarkers function in Seurat, filtering all markers based on FDR p<0.05, and using the resulting list of marker genes as the input for Binding Analysis for Regulation of Transcription (BART, v2.0). The resulting transcription factors were filtered based on a z-score >1 and Irwin-Hall corrected p-value <0.01.

### Bulk RNAseq

RNA was extracted via homogenisation using a bead beater, and the Qiagen RNEasy Plus Mini Kit. RNA was quantified using the Qubit v3 fluoremeter and the broad range RNA assay. Unique Molecular Identifiers (UMI) were incorporated (Twist UMI System) during library preparation. Sequencing was carried out on an Illumina NovaSeq (2x150bp, 20 million read pairs per sample). Sequencing QC (adapter trimming and deduplication based on UMI sequences) was carried out using fastp. Transcript counts were obtained using the package Salmon (v1.10.2), mapping to the human transcriptome (gencode v44, based on GRCh38.p14). Bulk RNAseq data from healthy liver tissue was obtained from two studies [9,10], available from the European Nucleotide Archive under accession numbers PRJNA523510 and PRJNA542148 respectively. Study samples were all sequenced in a single batch, and CombatSeq (within the R package sva v3.52.0) was used to correct for batch effects between study samples and the publicly available data.

Fold changes between experimental groups were calculated and used for gene set enrichment analysis using the package clusterProfiler (v4.12.6). against the Kyoto Encyclopedia of Genes and Genomes [11] and Gene Ontology (GO) [12] databases. The top enriched pathways/GO terms are presented for each condition, relative to healthy liver tissue.

### Cytokine profiling

Plasma samples were used to measure circulating cytokines. Samples were analysed using the Inflammation 20-plex and Immuno-Oncology 14-plex Human ProcartaPlex panels (Invitrogen), running on a Bio-Plex 200 system (Bio-Rad). The inflammation panel was used for all sample groups, the immuno-oncology panel was used for only pre and post-CPI and ChILI patients. Where available, patients recruited at baseline who went on to develop ChILI were separated from the Cancer pre-CPI group to measure the predictive effect of inflammatory cytokines (ChILI pre-CPI). Statistical comparison between groups was performed using the Kruskal-Wallis H test and adjusted for multiple comparisons using Bonferroni correction. Pairwise comparisons were made using the Mann-Whitney U test on cytokines shown to be significant by Kruskal-Wallis H test.

### Immunofluorescence

5 biopsies from checkpoint-induced liver injury patients were available, and compared to 5 acute DILI and 5 acute AIH biopsies. Formalin fixed paraffin embedded (FFPE) sections were cut to 4μm and sections were stained with antibodies to E-cadherin, CD8, CD38 and CXCR3, DAPI was used as a nuclear stain. Antibodies and fluorophores are included in supplementary table 4. TrueVIEW autofluorescence quencher (Vector Laboratories) was used prior to mounting (VECTASHIELD Vibrance media containing DAPI counterstain, Vector Laboratories). Sections were imaged using a Zeiss LSM780 confocal microscope. CD8+ cells, and CD8+CD38+CXCR3+ cells were counted manually using QuPath v0.4.3 [13].

### Code availability

No custom code was used for the analyses in this manuscript. With the exception of FlowJo v10, all software used is freely available: batchtma (v0.1.6), <https://stopsack.github.io/batchtma>; BART (v2.0) <https://github.com/zanglab/bart2>; CATALYST (v1.18.1), <https://bioconductor.org/packages/release/bioc/html/CATALYST.html>; clusterProfiler (v4.12.6), <https://bioconductor.org/packages/release/bioc/html/clusterProfiler.html>; ConsensusClusterPlus (v1.58.0), <https://bioconductor.org/packages/release/bioc/html/ConsensusClusterPlus.html>; CyTOFClean (v1.0.3), <https://github.com/JimboMahoney/cytofclean>; DESeq2 (v1.44.0), <https://bioconductor.org/packages/release/bioc/html/DESeq2.html>; diffcyt (v1.14.0), <https://github.com/lmweber/diffcyt>; fastp (v 0.23.4), <https://github.com/OpenGene/fastp>; FlowSOM (v2.2.0), <https://bioconductor.org/packages/release/bioc/html/FlowSOM.html>; limma (v3.64.1), <https://bioconductor.org/packages/release/bioc/html/limma.html>; QuPath (v0.5.1), <https://qupath.github.io>; Salmon (v1.10.2), <https://github.com/COMBINE-lab/salmon>; Seurat (v5.3.0), <https://cran.r-project.org/web/packages/Seurat/index.html>; slingshot (v2.2.1), <https://github.com/kstreet13/slingshot>; sva (v3.52.0), <https://bioconductor.org/packages/release/bioc/html/sva.htmll>; speckle (v1.8.0), <https://www.bioconductor.org/packages/release/bioc/html/speckle.html>

##

## References

1. Aithal GP, Watkins PB, Andrade RJ, et al. Case definition and phenotype standardization in drug-induced liver injury. Clin Pharmacol Ther 2011;89:806–815.

2. Mack CL, Adams D, Assis DN, et al. Diagnosis and Management of Autoimmune Hepatitis in Adults and Children: 2019 Practice Guidance and Guidelines From the American Association for the Study of Liver Diseases. Hepatology 2020;72:671–722.

3. Nowicka M, Krieg C, Crowell HL, et al. CyTOF workflow: differential discovery in high-throughput high-dimensional cytometry datasets. F1000Res 2019;6:748.

4. Van Gassen S, Callebaut B, Van Helden MJ, et al. FlowSOM: Using self-organizing maps for visualization and interpretation of cytometry data. Cytometry Part A 2015;87:636–645.

5. Wilkerson MD, Hayes DN. ConsensusClusterPlus: a class discovery tool with confidence assessments and item tracking. Bioinformatics 2010;26:1572–1573.

6. Weber LM, Nowicka M, Soneson C, et al. diffcyt: Differential discovery in high-dimensional cytometry via high-resolution clustering. Commun Biol 2019;2:1–11.

7. Street K, Risso D, Fletcher RB, et al. Slingshot: cell lineage and pseudotime inference for single-cell transcriptomics. BMC Genomics 2018;19:477.

8. Hao Y, Hao S, Andersen-Nissen E, et al. Integrated analysis of multimodal single-cell data. Cell 2021;184:3573-3587.e29.

9. He L, Davila-Velderrain J, Sumida TS, et al. NEBULA is a fast negative binomial mixed model for differential or co-expression analysis of large-scale multi-subject single-cell data. Commun Biol 2021;4:1–17.

10. Bankhead P, Loughrey MB, Fernández JA, et al. QuPath: Open source software for digital pathology image analysis. Sci Rep 2017;7:16878.
