# Supplementary material for: Circulating exhausted CD8+ effector memory cells differentiate immune checkpoint inhibitor-induced liver injury from other acute immune-mediated liver injuries
Source: J Immunother Cancer. 2026 Mar 27;14(3):e014178. doi: 10.1136/jitc-2025-014178 (PMC13034233; doi:10.1136/jitc-2025-014178)

Circulating exhausted CD8$^+$ effector memory cells differentiate immune checkpoint inhibitor-induced liver injury from other acute immune-mediated liver injuries

Authors

Stuart Astbury, Edmond Atallah, Jane I Grove, Amber G Bozward, Scott P Davies, Mark J Sheehan, Steven W Kumpf, Jessie Qian, Natalia M Krajewska, Grace E Wootton, Melanie R Lingaya, Davor Kresnik, Flavia Radulescu, Ankit Rao, Hester Franks, Lourdes Ruiz-Ortega, Mar Riveiro-Barciela, Shashi K Ramaiah, Thomas A Lanz, Changhua Ji, Poulam M Patel, Ye H Oo, Guruprasad P Aithal

Correspondence

stuart.astbury@nottingham.ac.uk; guru.aithal@nottingham.ac.uk

In brief

Using mass cytometry for broad immune phenotyping, we have identified a unique subset of CD8$^+$ T-cells that are expanded in immune checkpoint-inhibitor induced liver injury (ChILI). This subset was validated in an independent cohort, correlates with ALT, and bulk RNAseq demonstrates the immune response to these cells in the livers of ChILI patients. This cell subset has potential to differentiate ChILI from other competing diagnoses, and may aid in the stratification of ChILI patients for corticosteroid treatment,

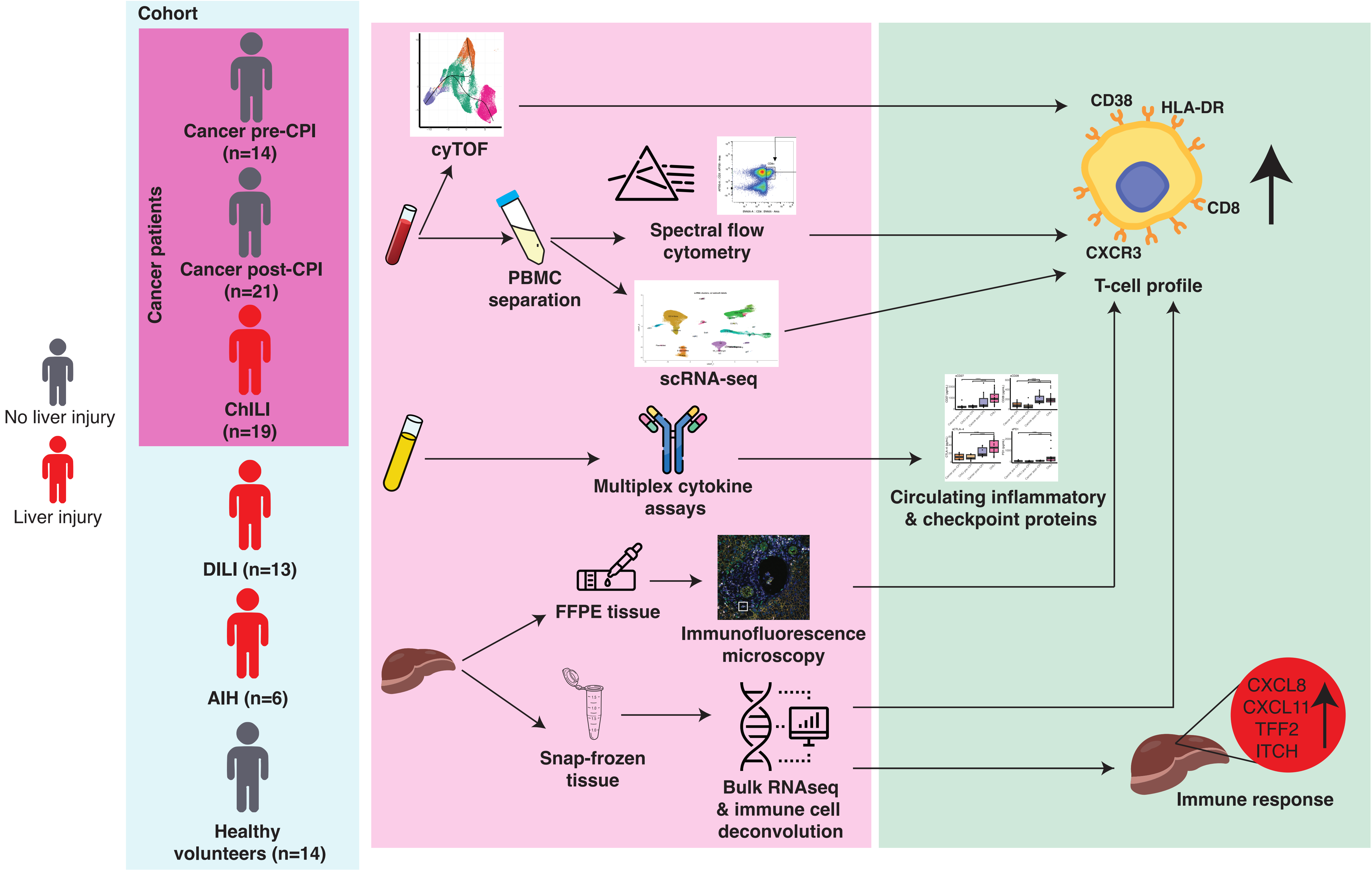

Supplement: online supplemental file 3 [file jitc-14-3-s003.pdf]
